# Creative Activities in Music – A Genome-Wide Linkage Analysis

**DOI:** 10.1371/journal.pone.0148679

**Published:** 2016-02-24

**Authors:** Jaana Oikkonen, Tuire Kuusi, Petri Peltonen, Pirre Raijas, Liisa Ukkola-Vuoti, Kai Karma, Päivi Onkamo, Irma Järvelä

**Affiliations:** 1 Department of Medical Genetics, University of Helsinki, Helsinki, Finland; 2 Sibelius Academy, University of the Arts Helsinki, Helsinki, Finland; 3 Conservatory of Joensuu, Joensuu, Finland; 4 Department of Biosciences, University of Helsinki, Helsinki, Finland; University of Georgia, UNITED STATES

## Abstract

Creative activities in music represent a complex cognitive function of the human brain, whose biological basis is largely unknown. In order to elucidate the biological background of creative activities in music we performed genome-wide linkage and linkage disequilibrium (LD) scans in musically experienced individuals characterised for self-reported composing, arranging and non-music related creativity. The participants consisted of 474 individuals from 79 families, and 103 sporadic individuals. We found promising evidence for linkage at 16p12.1-q12.1 for arranging (LOD 2.75, 120 cases), 4q22.1 for composing (LOD 2.15, 103 cases) and Xp11.23 for non-music related creativity (LOD 2.50, 259 cases). Surprisingly, statistically significant evidence for linkage was found for the opposite phenotype of creative activity in music (neither composing nor arranging; NCNA) at 18q21 (LOD 3.09, 149 cases), which contains cadherin genes like *CDH7* and *CDH19*. The locus at 4q22.1 overlaps the previously identified region of musical aptitude, music perception and performance giving further support for this region as a candidate region for broad range of music-related traits. The other regions at 18q21 and 16p12.1-q12.1 are also adjacent to the previously identified loci with musical aptitude. Pathway analysis of the genes suggestively associated with composing suggested an overrepresentation of the cerebellar long-term depression pathway (LTD), which is a cellular model for synaptic plasticity. The LTD also includes cadherins and AMPA receptors, whose component GSG1L was linked to arranging. These results suggest that molecular pathways linked to memory and learning via LTD affect music-related creative behaviour. Musical creativity is a complex phenotype where a common background with musicality and intelligence has been proposed. Here, we implicate genetic regions affecting music-related creative behaviour, which also include genes with neuropsychiatric associations. We also propose a common genetic background for music-related creative behaviour and musical abilities at chromosome 4.

## Introduction

Creative activities in music represent cognitive functions of the human brain. A creative performance can be defined as a production of work or performance that is both original and appropriate for the situation in which it occurs [1–3]. Creativity requires the presence of several traits including intelligence, perseverance, unconventionality, openness and the ability to think in a particular manner [3, 4]. Creative productivity, in general, can have large-scale societal and economic consequences. The ability to be creative needs cognitive skills that may reflect evolutionary advantage [5]. Creativity can either be considered as psychological creativity that has value at an individual level, or as historical creativity that has value in time [6]. In the art of music, historical creativity considers, for example, composers whose compositions are widely known. However, creative activities in music can also be small-scale individual activities. We have studied self-reported creative activities in music that can be considered as individual-level musical creative performances.

Creative activities in music (composing, arranging, improvising and other activities like conducting) can also be considered as a component of musical aptitude (see, e.g. Levitin [7]). Musical abilities comprise a wide variety of skills. These abilities may be based on different genetic predispositions but they may also share some common features [8].

Compositional processes have been studied, and composition research has been based on various theoretical foundations (for a review, see Pohjannoro [9]). However, to our knowledge there are no widely used tests to measure compositional activities even though measures of musical creativity exist (e.g. Gordon [10]; Wang [11]; Webster [12]). These measures have different kinds of activities, from asking the participant to produce as many different sounds as possible by using one sound source to improvising and composing. Often they are only suitable for certain age groups. Parts of measures of creativity (e.g. The Torrance Test of Creative Thinking [13]) have also been used for measuring musical creativity [14]. Improvisation is one sector of creativity. It is connected to musical performance, and it is particularly used in baroque and jazz music. It has also been widely used in music therapy (for a review, see Backer & Sutton [15]).

The biological background of creativity and insight in music have been studied via neuroimaging experiments: using functional magnetic resonance imaging (fMRI) during composing [16], completing an incomplete melody [17] and improvising musical pieces [18–20]. These studies have shown that several brain regions including the medial prefrontal cortex, premotor areas and the auditory cortex are linked to the improvisation or composition of music (for a review, see Dietrich and Kanso [21]). A fMRI study linked lyric improvisation to the prefrontal cortex, language and motor control regions and the amygdala [22]. Notably, neural activity in the auditory cortical areas can occur in the absence of sound [23] and music can be composed without hearing, whereas aphasia and apraxia can disturb composing [24]. There is evidence that the imagery of familiar melodies is strong enough to cause a pre-attentive brain response using imagery mismatch negativity (iMMN) in musicians, but not in non-musicians [25]. While neuroimaging studies can show which regions of the brain are active during creative tasks, the biomolecular background of human creative functions in music is largely unknown.

The molecular genetic basis of overall creativity has mostly been investigated with candidate gene approaches. Several genetic studies have linked dopamine with divergent thinking, which is a process of generating creative solutions and includes components like fluency, originality and flexibility [1]. The dopamine receptor *DRD2* has been linked to ideational fluency [26, 27] and its expression in the thalamus has been shown to be correlated with divergent thinking [28]. Other dopamine-related genes like *DRD4*, *DAT*, *TPH1* and *COMT* have been shown to be associated with divergent thinking, especially with fluency [26, 27, 29]. Some non-dopamine related genes have also been linked to creativity. A biologically relevant SNP in the promoter region of *NRG1* has been linked to originality, flexibility and fluency in a study of intellectual people [30]. Creative dance performance (with music) has been linked to *SLC6A4* and *AVPR1A* [4]. However, the authors hypothesised that these two genes might be involved in the social and emotional aspects of the performance, not creativity *per se*. It is of note that many of the candidate genes for creativity are also reported as risk genes for psychiatric diseases. Especially, genes responsible for dopamine and serotonin metabolism have been associated with both psychiatric diseases and creativity [27, 31]. Likewise, the SNP in the *NRG1* promoter region has been related to elevated psychosis risk [30]. Also, psychiatric disorders have been linked to creativity in population studies: schizophrenia, bipolar disorder, anorexia and autism have been found to be more common among relatives of individuals with creative professions including composers and other musicians [32].

We and others have performed candidate gene and genome-wide linkage and association studies on music-related traits [33–36]. These studies have shown that music-related traits are linked to many genes and genomic regions [33–36]. To our knowledge, no genome-wide analyses have been carried out for creative activities in music.

We have previously shown that self-reported creative activities in music (composing, arranging and improvising) have relatively high heritabilities: 0.40–0.84 [37]. Here, we used a larger sample and more detailed questionnaire to study the genetic basis of three different creative activities in music, composing, arranging and improvising, as well as the prevalence and genetic basis of other, non-music related creativity. In order to elucidate the molecular genetic background of creativity in music, we performed a genome-wide linkage and linkage disequilibrium analysis on the music-related creative activities and other, non-music related creativity.

## Materials and Methods

### Ethics statement

The study was approved by The Ethical Committee of Helsinki University Central Hospital (TMK03/233/13/03/03/2013) and was conducted in accordance with the Declaration of Helsinki. A written informed consent was obtained from all the subjects.

### Participants

The study included 577 participants who were previously studied in a genome-wide analysis of musical aptitude [35]. Of them, 5 did not have genotypes and 12 had not answered the questionnaire. Of the participants, a total of 474 individuals were familial belonging to 79 families and 103 were unrelated.

### Questionnaire

The creative activities in music and background information of the participants were collected using a self-reported questionnaire. As stated above, creative activities were defined as composing, arranging and improvising. Concerning the creative activities in music, the terms were first explained to the participants. The participants were asked if they did or used to compose, arrange and/or improvise music (S1 Table). From those individuals who were or had been active in arranging, composing or improvising, we collected more comprehensive information on the activity and related music pieces. Also, information about musical aptitude (the scores from three musical aptitude tests), all of which measure sensory-level processing and minimize the effects of culture, was available from 568 participants [35]. We also had information about the participants’ music education, practising and other musical activities [35, 37].

Additionally, all the participants were asked if they considered themselves to be creative in some other field. This other, non-music-related creativity included visual, scientific, technical, physical or verbal creativity, as explained to the participants (S1 Table). Moreover, the participants could position themselves in four classes of creativity: “Very creative”, “Quite creative”, “Slightly creative” or “Not at all creative”.

### Phenotype processing

#### Composing, arranging and improvising

A total of 103, 120 and 178 participants reported that they composed, arranged and improvised respectively and were classified as cases in the linkage and association analyses. In order to improve the reliability of the phenotypes, individuals who reported no activity in composing, arranging or improvising were included in the study only if they had at least two years of music education or had practised music at least for two years. This restriction ensured that individuals who answered “No” did it as a choice rather than lack of opportunity. Thus, the individuals who answered “No” and had practised music for less than two years were excluded from the composing, arranging and improvising control groups (189, 193 and 158 respectively).

#### NCNA

A combined phenotype was formed from the participants who reported that they neither composed nor arranged despite being musically educated. The individuals who neither composed nor arranged (NCNA) were denoted as cases, and the individuals who reported composing or arranging were denoted as controls for this phenotype. In total the NCNA phenotype included 149 cases and 159 controls.

#### Other creativity

The non-music-related creativity (other creativity) was studied separately to identify whether the results for the music-related traits were music-specific or related to any kind of creativity. The other creativity was dichotomized for the genetic analyses: The cases included individuals who had answered that they were “Very creative” or “Quite creative” (S1 Table). The control group (referred also as the “No” group) comprised of individuals who reported themselves as “Not at all creative” or “Slightly creative”. Naturally, other creativity was not restricted to music-educated or practised individuals. Thus, there were additionally 131 individuals who were not included in the music-related analyses. Overall, there were 259 creative individuals and 162 individuals in the control group.

In total 473 of the participants had at least one accepted phenotype. There was some overlap between the phenotypes. There were 50 individuals who reported being creative in arranging, composing and other, non-music-related fields (S1 Fig).

### Heritability

Heritabilities were evaluated from the family material using the DMU program (version 6.5.2 [38]) with the logit link model. Age was used as a covariate and gender as a fixed effect in the evaluation.

### Laboratory procedures

DNA was extracted from peripheral blood as explained in Oikkonen *et al*. [35]. The samples were genotyped with the Illumina HumanOmniExpress 12 1.0V SNP chip (www.illumina.com) that contains over 700,000 SNPs. More information about the genotyping is available in Oikkonen *et al*. [35].

### Quality control

Quality control was performed with PLINK 1.07 [39]. Relatedness was evaluated with the identity by descent calculations. In total, three subjects were removed due to incorrect relatedness. No individuals were removed for missing genotypes (>5%).

Markers were removed for missingness (>5%), Mendelian inconsistencies, Hardy-Weinberg equilibrium (p-value <0.001) and low minor allele frequency (<5%). A total of 590,979 markers remained, which were all used for the linkage disequilibrium analysis.

Population stratification was studied with principal component analyses with EIGENSOFT 6.0.1 using unrelated individuals. No population stratification was seen between the cases and the controls in any of the phenotypes (S2 Fig).

### Multipoint linkage

Linkage analyses were performed with Merlin (v1.1.2) [40] as non-parametric analyses. SNPs were pruned to exclude linked and uninformative SNPs. First, the minor allele frequency was required to be >0.25 to maximize the information per selected SNP. Second, SNPs in high linkage disequilibrium (LD) with each other were removed. LD pruning was performed with the variant inflation factor method in PLINK with a variant inflation factor limit of 1.25 (corresponding to an LD of 0.2 as measured by R^2^). Third, the remaining SNPs were pruned for a map distance of 0.2cM (Rutgers map v.2 [41]). Finally, the remaining SNPs were rerun for Mendelian errors with PedCheck [42]. In total, 10,826 SNPs were included in the linkage analyses.

**Fig 1:**
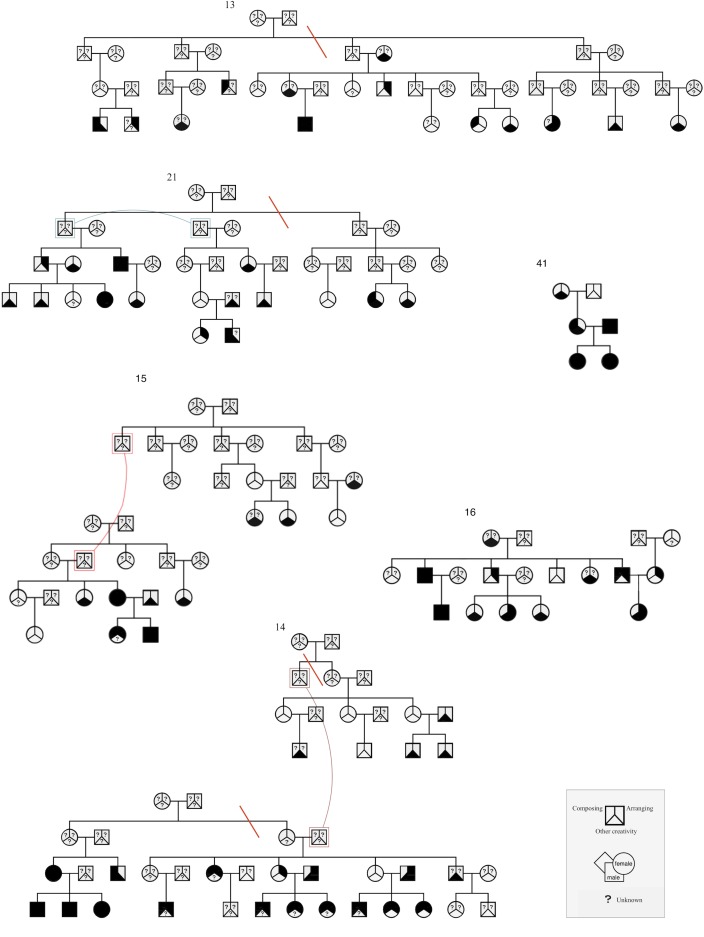
Examples of the pedigrees in the study. Arranging, composing and other creativity answers are shown for every individual; black denotes “yes” and white “no”. The question marks denote missing phenotypes. Individuals shown twice are linked with a coloured curve. Orange lines mark where the three most complex pedigrees (#13, #14 and #21) were split for the Merlin linkage analyses. The complexity is caused by the number of individuals and missing phenotypes in a pedigree. In some of the families, as in #14 and #41, music-related creative activities were common, whereas most of the families showed only a few individuals who arranged and/or composed music.

Each of the binary traits: composing, arranging, other creativity and NCNA, were analysed separately. After removing sporadic individuals and uninformative family members, linkage data included 467 genotyped individuals in 79 families. For Merlin, the largest families were split to have a maximum of 25-bit complexity: families 13 and 21 were split into two parts and family 14 into three parts (Fig 1).

### Linkage disequilibrium

Genome-wide linkage disequilibrium analyses were performed at two stages with all the genotyped and phenotyped individuals. The numbers of individuals with approved phenotypes are shown in Table 1. All analyses included 572 genotyped individuals. First, all SNPs were analysed with Plink with the case-control association analysis option to select SNPs for further analysis. This analysis option did not consider the family structures. Next, Pseudomarker 2.0 [43] was used for more complex linkage disequilibrium (LD) analysis. Pseudomarker can utilize both the families and sporadic individuals and reconsider the available family structures. Because it is computationally intensive, only the nominally associated SNPs from the PLINK analyses (p-value≤0.01) and the SNPs within linked regions (peak LOD≥2.0, encompassing regions around the peaks until LOD score drops by 1.0) were analysed. From 590,979 markers in the first phase, 14,135, 13,069, 11,875 and 10,749 markers were analysed successfully in the second phase respectively for arranging, composing, NCNA and other creativity.

**Table 1:** Numbers of individuals and heritabilities in each phenotype. The heritabilities were estimated from the family material. The heritability estimates (H_2_R) are accompanied by p-values and standard errors (SE).

	Yes		No		Total	Heritability		
	N	%	N	%	N	Genetic variance	SE	Heritability
Composing*	103	36.0	183	64.0	286	0.499	0.382	0.333
Arranging*	120	42.3	164	57.7	284	0.502	0.367	0.334
NCNA*			149	48.4	308	0.407	0.348	0.289
Improvising*	178	62.9	105	37.1	283	0.131	0.344	0.116
Other creativity	259	61.5	162	38.5	421	0.520	0.290	0.342

* Individuals in the “No” group were only included, if they had at least two years of music education or practice of music to ensure the credibility of the answers (see Materials and Methods, Phenotype processing).

Pseudomarker allows one liability factor to be incorporated. The phenotypic correlations were analysed to choose the liability factor. Age and gender were found to correlate with the traits. We used age as the liability factor, divided into two groups: ≤32 years and >32 years (S3 Fig). As only one factor could be considered, age was chosen over gender, as we wanted to correct for the most obvious environmental sources of heterogeneity. Musical aptitude scores were also different between creative and non-creative individuals but these were considered as part of the phenotype and thus we did not want to correct for it.

Pseudomarker performs joint linkage and LD, two-point linkage, LD given linkage and LD given no linkage tests. The joint linkage and LD was considered the most suitable for our mixed data. All Pseudomarker analyses were performed with both recessive and dominant models.

### Genome-wide significance levels

The linkage results are considered significant if the LOD score is at least 3.0 and suggestive when above 2.0 [44]. In genome-wide association studies, results with a p-value below 5x10^-8^ are usually considered significant, which we applied for linkage disequilibrium analysis. However, we implemented 2-phase linkage disequilibrium analysis where the use of joint linkage and LD might loosen the correction level needed to correct for multiple testing as it also uses information from the linkage. Thus, we additionally considered results better than 5x10^-6^ as suggestive and interesting.

### Functional analysis of the associated genes

The joint linkage and LD results were further studied with Ingenuity Pathway Analysis (IPA, QIAGEN Redwood City, www.qiagen.com/ingenuity, Spring 2015 release). All genes (RefSeq, hg18) 1Mb upstream and downstream of suggestively associated SNPs (joint p-value<1x10^-4^) were included in the pathway and function analyses. These thresholds resulted in 668, 672, 583 and 637 genes in the analyses for arranging, composing, NCNA and other creativity, respectively. Only annotations with at least 3 associated genes were considered.

## Results

### Characteristics of the phenotypes

The study enrolled 577 subjects including both families and sporadic individuals, where phenotype was available for 286, 284, 283 and 421 individuals for composing, arranging, improvising and other creativity respectively. The other creativity entails subjects’ own assessments of creativity in non-musical fields: visual, verbal, scientific, technical or physical. We also separately analysed the opposite phenotype of creative activity in music: music-experienced individuals, who were neither active in composing nor arranging (neither compose nor arrange; NCNA).

The general characteristics of the participants are shown in Table 2. One-third of the individuals who answered the questionnaire practised either composing or arranging. This high proportion could be due to the fact that there are more musicians and active amateurs in our sample than in the overall population. Also, some large families included in the study have considerably large number of individuals who compose or arrange music (Fig 1).

**Table 2:** Characteristics of the phenotypes. Creative activities are associated with age, gender and musical aptitude scores (the Karma music test (KMT) and the Seashore test for pitch discrimination (SP)). Non-music-related creativity is independent of gender, but dependent of age and musical aptitude scores. We used a two-sided t-test for continuous variables and a two-sided Fisher’s exact test to test the difference between males and females.

Trait		Mean age	Mean KMT scores	Mean SP scores	Prevalence, % (N)	
					Males	Females
Composing	Yes	32.7	36.4	46.0	45.0 (59)	31.8 (62)
	No	41.7	34.3	45.1	55.0 (72)	68.2 (133)
	*p-value*	*<0*.*001*	*<0*.*001*	*0*.*084*	* *	*0*.*019*
Arranging	Yes	35.3	36.8	46.4	53.5 (68)	33.8 (66)
	No	40.9	33.9	44.8	46.5 (59)	66.2 (129)
	*p-value*	*0*.*004*	*<0*.*001*	*0*.*001*	* *	*0*.*001*
NCNA		42.0	33.8	44.7	38.4 (53)	57.4 (117)
	*p-value*	*<0*.*001*	*<0*.*001*	*0*.*010*	* *	*0*.*001*
Improvising	Yes	38.5	34.7	44.7	72.2 (78)	57.1 (100)
	No	44.2	33.6	45	27.8 (30)	42.9 (75)
	*p-value*	*0*.*008*	*0*.*096*	*0*.*537*	* *	*0*.*012*
Other creativity	Yes	40.3	33.1	43.5	62.3 (109)	62.3 (174)
	No	44.5	31.9	42.1	37.7 (66)	37.7 (104)
	*p-value*	*0*.*020*	*0*.*048*	*0*.*027*	* *	*1*.*000*
Total material		42.6	35.1	45.4	40.4 (191)	59.6 (282)

In our data, composing, arranging and improvising were more common among males than females (Table 2). However, there were no differences in other fields of creativity between males and females. The gender differences in creative activities in music were the most evident in older age groups but appeared also among teenagers (S4 Fig). The level of music education between the subjects was similar for both genders.

The musical aptitude scores were relatively higher among composing and arranging individuals, as in our previous study [37]. Interestingly, the musical aptitude scores were also higher among individuals who reported creativity in other fields (Table 2).

### Heritability

Heritability is the proportion of the phenotype variation explained by additive genetic factors. The heritability estimates ranged from 29% to 34% for composing, arranging, NCNA and other creativity (Table 1). The heritability estimate for improvising was only 11% and it was thus left out from the following genetic analyses. These estimates are lower than the heritabilities published in our previous study by Ukkola *et al*. [37]. Here, we used a larger sample and more focused phenotypes that should produce more reliable estimates of heritability.

### Arranging

**Fig 2:**
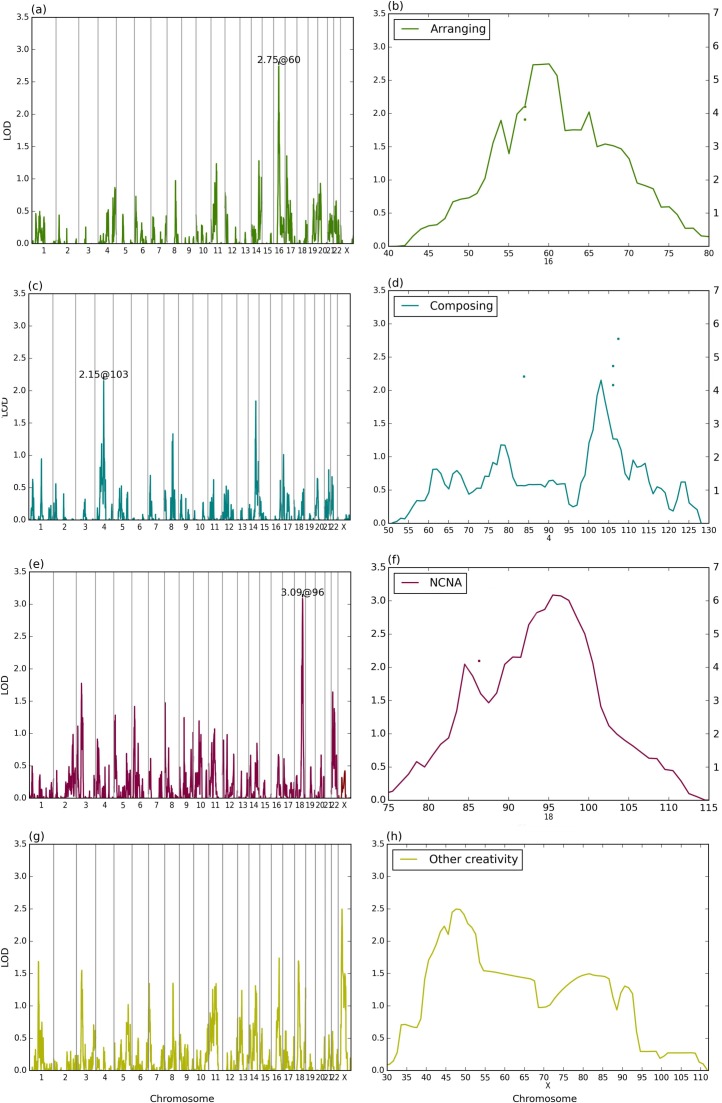
Merlin linkage results. Multipoint linkage LOD scores for arranging (a), composing (c) NCNA (e) and other creativity (g). The chromosomal region around the best LOD score is depicted for each phenotype (b, d, f and h for arranging, composing, NCNA and other creativity, respectively). On the best regions, multipoint linkages are illustrated by curves (scale on the left) and the best joint linkage and LD results by points as negative logarithmic values (scale on the right). The X-scale shows positions as centimorgans.

Self-reported arranging was linked to chromosome 16 (LOD score 2.75) (Fig 2). The region covers 16p12.1-q12.1 on both sides of the centromere. The best single-point linkage was obtained for rs9933639 located in the intron of the *GSG1L* gene at 16p12.1 (LOD 4.22, joint LD and linkage p-value 3.5x10^-5^). *GSG1L* is part of the AMPAR complex, which is an important transmembrane receptor for glutamate that mediates fast synaptic transmission in the CNS [45].

Concerning arranging, suggestive joint linkage and LD was found at 2p24.3 (p-value 1.7x10^-7^ at rs6531144; Table 3, S5 and S6 Figs) between the *FAM49A* and *MYCN* genes. Defects in *MYCN* cause Feingold syndrome type 1, which affects brain function including microcephaly, intellectual disability, digital malformations, and deafness [46]. The function of the *FAM49A* is not known, but it is paralog of *FAM49B*, which was found at the linked region in an absolute pitch study [33] and in a duplicated CNV region identified in a subject with low musical aptitude scores in our previous study [47]. Both genes are expressed in the human brain [48].

**Table 3:** Best joint LD and linkage results. Only the results with p-value < 5x10^-6^ are shown. The nearest genes were obtained from the RefSeq database and the main functions from UCSC (http://genome-euro.ucsc.edu/) and NCBI (http://www.ncbi.nlm.nih.gov/gene/). NA, not available; TF, transcription factor; lcRNA, long non-coding RNA; rec, recessive; dom, dominant

Region	Marker	Phenotype	Model	LOD	Joint p-value	Nearest genes	Main gene function
10q22.1	rs1417940	Other creativity	rec	1.90	1.5x10^-7^	*STOX1*	Pre-eclampsia
2p24.3	rs6531144	Arranging	rec	2.68	1.7x10^-7^	*FAM49A*	Paralog of *FAM49B*
						*MYCN*	TF, Feingold syndrome, neuroblastoma
2q31.1	rs4972527	Other creativity	rec	3.97	3.4x10^-7^	*RAPGEF4*	Energy metabolism, Ras signalling pathway
6q15	rs1528983	NCNA	dom	0.34	9.3x10^-7^	*MAP3K7*	Cell response to environmental stress
7p21.1	rs518136	Composing	dom	0.36	1.0x10^-6^	*AGR3*	Breast cancer
						*AHR*	TF, aromatic hydrocarbon effects
3p14.3	rs1375515	Other creativity	dom	0.12	1.0x10^-6^	*CACNA2D3*	Brain specific calcium-channel
20q13.2	rs1293430	NCNA	dom	0.08	2.0x10^-6^	*TSHZ2*	TF, cancer
8p23.2	rs4242493	Composing	rec	0.50	2.0x10^-6^	*CSMD1*	Carcinoma suppressor
4q22.3	rs10856917	Composing	dom	1.87	2.0x10^-6^	*UNC5C*	Cell migration, axon extension
14q13.1	rs737401	Composing	dom	2.56	2.0x10^-6^	*EGLN3*	Oxygen censor, hypoxia, pre-eclampsia
						*NPAS3*	TF, possibly neurogenesis, schizophrenia
16p12.1	rs4073229	Other creativity	rec	0.08	2.0x10^-6^	*HS3ST4*	Brain specific sulfotransferase
14q21.3	rs2225994	NCNA	rec	0.02	4.0x10^-6^	*LINC00648*	lcRNA
4p14	rs4833107	NCNA	dom	0.18	4.0x10^-6^	*ARAP2*	Actin cytoskeleton remodelling
						*DTHD1*	Signalling, apoptosis
12p12.2	rs2728554	Arranging	rec	0.00	4.0x10^-6^	*LOC100506393*	lcRNA
6q25.3	rs9397906	Other creativity	rec	1.85	4.0x10^-6^	*NOX3*	Inner ear structures
						*ARID1B*	Neural development and dendrite growth
17q11.2	rs8064530	Other creativity	dom	3.47	4.0x10^-6^	*PIPOX*	NA
						*SEZ6*	Neuronal membrane signalling
7q34	rs12667802	Arranging	dom	1.87	4.0x10^-6^	*PRSS37*	Protease
						*OR9A4*	Olfactory receptor
						*CLEC5A*	Inflammatory response

Functional analysis of all the genes suggesting an association in joint LD and linkage analysis with arranging showed an overrepresentation of X-linked autism (4 genes, p-value 2.1x10^-6^) and X-linked hereditary disease (25 genes, p-value 4.0x10^-6^). The X-linked autism genes included *MECP2*, *NLGN3*, *RPL10* and *TMHLE*. All overrepresented functions and their associated genes can be found in S2 Table.

### Composing

**Fig 3:**
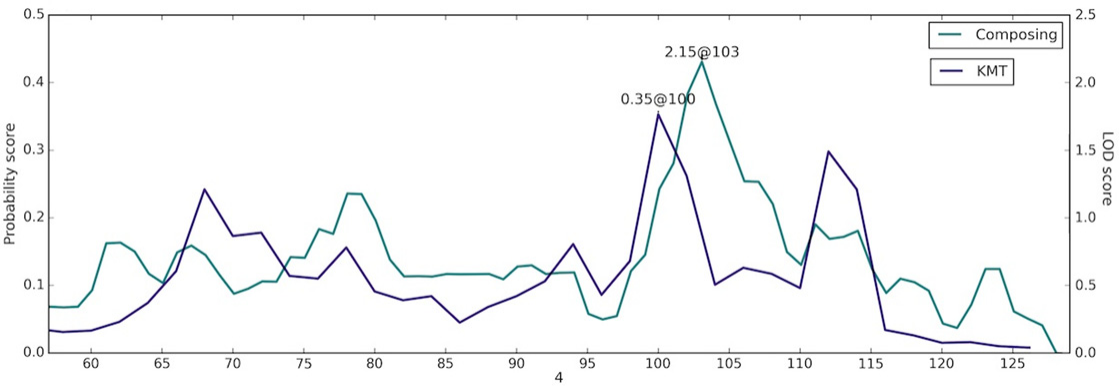
Chromosome 4 linked region overlaps with our previous finding with musical aptitude. Composing is linked to 4q22.1. In a previous article, the same region was found to be linked with KMT scores [35]. The linkage results are shown with solid lines, composing results on the LOD score scale (axis scale on the right) and KMT results as probability scores (scale on the left). The position information is shown in centimorgans.

Self-reported composing showed some evidence for linkage to chromosome 4q22.1 with a multipoint LOD score of 2.15. Notably, this region overlaps with our previous findings for musical aptitude (Fig 3) [35, 49]. The best result for joint linkage and LD in the region was found on 4q22.3 at rs10856917, which resides in the *UNC5C* gene intron (p-value 2x10^-6^). Netrins, to which UNC5C belongs, are secreted proteins that direct axon extension and cell migration during neural development [50]. Nearby the linked region, there is also the *UGT8* gene (at 4q26) that was found to be associated with musical ability in Mongolian families [36] suggesting a shared molecular background for musical aptitude and composing.

**Fig 4:**
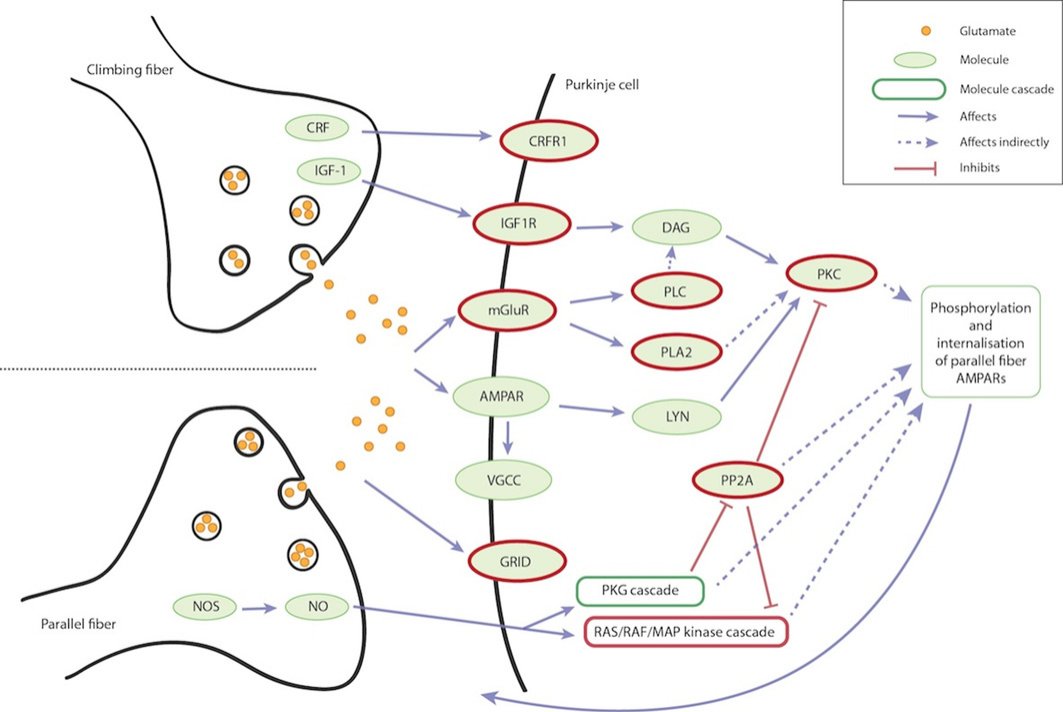
Synaptic long-term depression pathway. The LTD pathway was found to be overrepresented among genes suggestively associated with composing. This diagram shows the main molecules and their relationships that are involved in the LTD process. The climbing fibre originates from inferior olive and parallel fibres from the cerebellar cortex. There are various parallel fibres in contact with each Purkinje cell. Stimuli from climbing and parallel fibre impact Purkinje cell partially through the same receptors (mGluR and AMPAR). Parallel fibres function also through NO and GRID; the climbing fibre through CRFR1 and IGF1R. The climbing and parallel fibres are here shown side by side, but they may reside on different parts of the Purkinje cell. The pathway influences phosphorylation and internalization of AMPAR in parallel fibre, which in turn affects the synaptic function for future stimuli. The molecules and cascades related to genes that were suggestively associated with composing are lined with red (see Table 4).

The functional analysis of the joint linkage and LD results for composing revealed an overrepresentation of the cerebellar synaptic long-term depression (LTD) pathway (p-value 9.0x10^-8^; Fig 4). The LTD pathway changes synaptic efficacy to subsequent stimulus. A total of 141 genes are known to be involved in the LTD pathway (IPA, QIAGEN Redwood City, www.qiagen.com/ingenuity), of which 17 genes across ten genomic regions were found in our joint LD and linkage analysis (Table 4, Fig 4). Interestingly, this pathway also includes AMPA receptor (AMPAR). Several forms of AMPARs exist, and interestingly, one of them includes GSG1L that was linked with arranging (see above).

**Table 4:** Genes involved in LTD pathway. The LTD pathway was found to be overrepresented in the genes pinpointed by our joint LD and linkage analysis of composing.

Region	p-value	SNP	Gene	Part of molecule or cascade in LTD pathway
1p36	7.0x10^-6^	rs11799849	*PLA2G5*	PLA2
			*PLA2G2A*	PLA2
			*PLA2G2C*	PLA2
			*PLA2G2D*	PLA2
			*PLA2G2E*	PLA2
			*PLA2G2F*	PLA2
1p13.2	3.6x10^-5^	rs10801911	*NRAS*	RAS: RAS/RAF/MAP kinase cascade
1p13.1	9.6x10^-5^	rs639208	*PPM1J*	PP2A
2p21	2.7x10^-5^	rs4233921	*PRKD3*	PKC
3p26.1	7.2x10^-5^	rs163968	*GRM7*	mGluR
4q22	1.4x10^-5^	rs1905724	*GRID2*	GRID
9q34	6.4x10^-5^	rs11793700	*PPP2R4*	PP2A
10p15	7.0x10^-5^	rs12357847	*PRKCQ*	PKC
11p15.2	6.6x10^-5^	rs10766117	*RRAS2*	RAS: RAS/RAF/MAP kinase cascade
15q26.3	6.2x10^-5^	rs1223954	*IGF1R*	IGF1R
17q21.31	8.6x10^-5^	rs3744760	*CRHR1*	CRFR1
			*PLCD3*	PLC

Concerning composing, we also found suggestive evidence for joint linkage and LD at *CSMD1* intron (rs4242493, p-value 2.0x10^-6^), a locus that is also associated with schizophrenia [51].

### Neither composing nor arranging (NCNA)

Unexpectedly, we found evidence of linkage in the group of participants who did not compose or arrange but have practised music, on chromosome 18q21 (LOD 3.09), exceeding the genome-wide significance threshold. The best two-point linkage within the region was found at 18q22.1 near *CDH7* and *CDH19* genes (LOD 3.3), and the best joint LD and linkage was found at rs11152166 (p-value 5.1*10^−5^, dominant model), which has previously been linked to major depressive disorder [52].

Overall, the best joint linkage and LD for NCNA pinpointed rs1528983 near *MAP3K7* (p-value 9.3*10^−7^) at 6q15 (S7 Fig). The second best joint linkage and LD was at 20q13.2 (rs1293430, p-value 3.0*10^−6^) within the *TSHZ2* intron. Among these suggestively associated markers, rs2225994 (p-value 4.0*10^−6^) is located near a set of markers that have been suggestively associated with cognitive capabilities: cognitive performance [53, 54], social communication problems [55] and post-traumatic stress disorder [56].

### Self-reported other creativity

We did find suggestive evidence for linkage at Xp11.23 (LOD 2.50), for non-music related creativity (Fig 2). The linked region extends 20 cM and contains several genes (for example *SYN1*, *ELK1*, *KCND1* and *SYP)* that affect brain function. *Synapsin 1* (*SYN1*) is involved in synaptic vesicle clustering and recycling, neuronal development and synaptogenesis. Mutations in *SYN1* have been associated with epilepsy and autism spectrum disorder [57]. *ELK1* regulates genes expressed in the striatum, the reward centre of the human brain [58]. *KCND1*, a voltage-dependent Kv1.4 channel subunit is expressed in the hippocampus, olfactory bulb, and heart [59]. SYP is the most abundant synaptic vesicle membrane protein, which regulates synaptic vesicle endocytosis [60]. Mutations in *SYP* lead to X-linked intellectual disability implicating its involvement in learning and memory.

With other creativity, we found suggestive evidence in the joint linkage and LD scan for two genomic locations. At chromosome 10q22.1, SNPs rs10082399 and rs1417940 (best p-value 1.5x10^-7^) in intron region of STOX1 were suggested (Table 3, S8 Fig). At chromosome 2q31.1, SNP rs4972527 (p-value 3.4x10^-7^) was suggested. This SNP lies in an intron of *RAPGEF4* and near the *ZAK* gene (S9 Fig). *RAPGEF4* has been associated with autism and linked to behavioural flexibility in mice [61]. The suggestively linked region did not include any SNPs with significant p-values.

## Discussion

This study gives preliminary evidence for the molecular genetic background of creative activities in music. We found suggestive evidence for linkage at 16p12.1-q12.1 (LOD 2.75) for self-reported arranging and at 4q22.1 (LOD 2.15) for self-reported composing and significant evidence at 18q21 (LOD 3.09) for NCNA, the opposite phenotype of creative activity in music. Joint LD and linkage analysis including both families and sporadic individuals suggested that there are also multiple other genomic locations: the best were at 2p24.3 for arranging (p-value 1.7x10^-7^) and at 6q15 for NCNA (p-value 9.3x10^-7^). However, we were unable to tag significant SNPs within linked regions.

The most intriguing results were obtained in pathway analysis of the identified loci and their nearby genes. Pathway analysis suggested overrepresentation of genes involved in cerebellar LTD, with composing (p-value 9.0x10^-8^). LTD is considered to be a cellular model for synaptic plasticity and memory (for a review, see Collinridge *et al*. [62]). Neuronal activity regulates AMPA receptor (AMPAR) localization in a synapse via the LTD pathway and weakens the synapse transmission for subsequent stimulus. Thus, LTD facilitates the acquisition of novel information or elimination of previously learned information, which is compatible with the cognitive function needed in composing. The cerebellum is involved in motor and cognitive tasks [63]. In music, the cerebellum has an impact on musical abilities [64]; it has been shown to be active during improvisation [18, 20] and it may be involved in working memory for rhythm [65]. Recently, early childhood music training and timing performance were found to be correlated with a smaller cerebellar white matter volume reflecting synaptic plasticity [66]. All these brain studies show that the cerebellum is important for musical abilities including non-motor related tasks, thus supporting our findings.

Interestingly, the AMPAR relates also to arranging, where the best two-point linkage was found at *GSG1L*. The AMPAR complex consists of multiple proteins, and its compositions are brain region-specific [67]. *GSG1L* containing AMPAR complexes are enriched in the cortex and are also found in the hippocampus, striatum and the thalamus [67, 68]. These regions are known to be involved in music perception of emotions [69, 70]. Especially the role of cortex is interesting here, as prefrontal, premotor and auditory cortices have been linked to improvisation and composing (for a review, see Dietrich and Kanso [21]). Additionally, the AMPAR is important throughout the auditory pathway [71]. Interestingly, *NRG1* that has previously been shown to be associated with divergent thinking, also regulates cerebellar AMPARs activity-dependently [72]. Overall, the cerebellar LTD that also includes AMPAR and cadherins [73] is important in synaptic plasticity, memory and other cognitive abilities that are prerequisites for composing and arranging. Our results indicate that individual differences in genes affecting the LTD alter music-related creative behaviour.

To our surprise, NCNA yielded significant linkage on 18q21. The region includes numerous genes related to brain functions including *CDH7*, *CDH19* and *CDH20* from the cadherin family. Cadherins are calcium-dependent cell adhesion molecules. The *CDH7* gene and many other genes from the cadherin family have been associated with neuropsychiatric disorders like schizophrenia (see Redies *et al*. [74] for review). *CDH7* is also known to be involved in the song development of finch [75]. There is evidence that human music perception and practice share a common genetic background with the vocalization of songbirds [76, 77]. The cadherins may also be important in LTD through their interaction with β-catenin [73]. It is known that increased levels of β-catenin in the hippocampus weaken spatial memory flexibility and reversal learning in mice [73]. Overall, the linked region at 18q21 seems to be important for many neurodevelopmental disorders including bipolar disorder and depression, and is specifically known for 18q deletion syndrome (symptoms include intellectual disability, seizures and sometimes hearing loss (OMIM, http://www.omim.org)). Also, the region at 14q21.3 that was suggestively associated with NCNA includes alleles with cognitive and psychiatric implications [53–56]. On the other hand, genetic loci that are related to psychiatric disorders may have a positive impact on cognitive traits like creativity (for a review, see Carson [31]). Whether this is due to same or different alleles has not been explicitly described. In support of the former, the relatives of individuals in creative professions are more likely to have schizophrenia, bipolar disorder, anorexia nervosa and autism than the rest of the population [32]. We hypothesize that the 18q21 region might include genetic factors contributing to vulnerability, which may lead to psychiatric disorders or creativity depending upon the environment. The NCNA may appear as a contradictory phenotype for these. Furthermore, Xp11.23 that was suggestively linked with other creativity includes genes connected to neuropsychiatric disorders. Also, the suggestive joint linkage and LD result for other creativity at 2q31.1 was located at *RAPGEF4* that is known to affect cognitive abilities. Obviously, the relevance of these cognitive and personality linked genes in creativity related traits should be studied in detail.

To date, several genomic approaches have pinpointed chromosome 4q22-23 as the candidate region for music-related traits [35, 36, 49, 76, 77]. In our original linkage study, chromosome 4q22 gave the highest linkage values with musical aptitude (COMB scores) [49]. Later on, Park et al [36] assigned the region with the pitch perception accuracy (PPA) test in Mongolian families where the variants in the *UGT8* gene were significantly associated. More recently, we assigned the linkage to 4q22 with musical aptitude using a Bayesian approach [35]. Interestingly, the *α-synuclein gene* (*SNCA*) that was found to be one of the most up-regulated genes after listening to and performing music is also located at 4q22 [76, 77]. In this study, composing was tentatively linked to the same region (Fig 3). We also found suggestive linkage disequilibrium at the linked region at an intron of the *UNC5C* gene. This gene is a netrin receptor for axon guidance during neural development and may affect neuronal cell death in the adult nervous system [78]. In conclusion, this region might harbour genes that are involved in a broad range of musical abilities. Furthermore, the other two suggestively linked regions at 18q21 and 16p12.1-q12.1 are adjacent to the previously identified loci with musical aptitude. Both of these are located about 10 centimorgans from the musical aptitude linked regions. The implicated joint linkage and LD result near *FAM49A* for arranging is also interesting as there is evidence for its paralog *FAM49B* in other music-related phenotypes.

According to Salimpoor *et al*. [70] a composer "manipulates structural and temporal aspects of music" and these aspects are related to frequency, timing, intensity and timbre. Thus, good performance in these skills is vital for musical creativity. In our previous study, we had measured perception of frequency and duration differences (Seashore pitch, Seashore time) [79] and auditory structuring ability in sound sequences (KMT) using frequency, loudness, duration and timbre for creating patterns [80]. Clearly, the individuals active in composing and arranging outperformed the inactive individuals in these musical aptitude tests (Table 2). Interestingly, the creative individuals in fields other than music also performed better in these tests in our study. There is a correlation between performance in musical auditory discrimination tests and intelligence, which might result from a correlation between testing and cognitive tasks (e.g. short-term memory) [81]. Thus, creative tasks in music may correlate with cognitive capacities that are also related to intelligence. Indeed, creativity has been suggested to be a part of intelligence [30].

We found a difference in the prevalence of creative activities in music between genders and age groups. As noticed also by Vinkhuyzen *et al*. [82], the older participants were less inclined to report arranging or composing: 48% of teenagers either composed or arranged whereas only 13% of the over 60-year olds did/had done. This difference may reflect a cultural change, the altered attitude towards creativity or change in the willingness to report these kinds of activities. For example, the national curriculum for music education in Finland places greater emphasis on the role of composing and improvising more than before. It has been suggested that there can be gender differences concerning which creative activities an individual most probably performs [83]. Thus, women and men could be equally creative in music, but men compose and arrange more than women. The background of the gender difference can include both biological and environmental reasons. Especially sociocultural factors, like inequality and gender stereotypes, have been suggested to affect creative productivity and self-assessment of it [83, 84].

There are some limitations in this study. The phenotype was based on self-reported creativity in music, determined using a questionnaire, as no specific test to define the phenotype is available. There might be biases like dishonesty and diversity of the participants in the questionnaire answers as explained by Ukkola *et al*. [37]. Genetic factors contributing to self-reported musical aptitude have previously been shown in a twin study [82], which supports our approach to map genes for self-reported musical traits. The self-reported creative activity did not measure how well the individuals perform these activities, nor the quality of the outcome, instead it concentrated on implementation of musical creativity. Hence, the creative activities in music are related to a personal willingness and motivation that may reflect an innate desire. Notably, distinctive proportions of active amateurs and even professional musicians, consider themselves non-creative; our question was whether this is more biological, or is it simply learned? In order to compensate for the relatively small sample size, extended families were utilized to ensure genetic homogeneity that increases the possibility to find the major loci. As the creative phenotypes are complex, no strong single gene effects were presumed. As exemplified in other behavioural human phenotypes, there are supposedly hundreds of genes affecting music related creativity. As such, it was expected that most of the results were only suggestive. Moreover, there was a phenotypic correlation between the traits (S1 Fig), which means that the measured four traits represent fewer underlying traits. However, we did not correct for the multiple phenotypes. Thus, future studies should be performed to evaluate the findings with larger samples.

According to Levitin [7], musical ability manifests itself in various ways that might be uncorrelated with each other. To study the whole spectrum of musical ability, we need to consider multiple music-related traits. In this study as well as in our previous studies, we have studied the genetic background of some variability of this musical ability. Overall, musical traits [33–36] including also music-related creativity seem to be at least partially inherited. We propose some common genetic background especially with composing and other music related traits at chromosome 4. Supposedly, the various musical abilities also retain independent genetic impacts. Here, we suggest cerebellar memory function to be important in creative activities in music. Also, we pinpointed regions including genes and alleles with neuropsychiatric implications. In spite of the deleterious effects on the human mind, neuropsychiatric disorders have persisted in evolution suggesting that they perhaps also carry advantageous effects such as creativity.

## Supporting Information

S1 Fig Overlap between answers in composing, arranging and other creativity. The number of the “yes” answers for composing, arranging and other creativity are shown here with overlap between the answers. Over half of the “yes” answers between arranging and composing overlapped. Overall, 320 individuals arranged, composed or considered themselves to be creative.
(PNG)

S2 Fig Population stratification using principal component analysis. The population stratification was studied with principal component analysis using EIGENSOFT 6.0.1. Only unrelated individuals (founders or other unrelated individuals from each family) were used. Plots from the top two principal components are here shown for arranging (A), composing (B), NCNA (C) and other creativity (D).
(PNG)

S3 Fig **Histograms of age among active and passive subjects in arranging (A) and composing (B).** Frequency of the individuals who compose or arrange drops after the age of 32. This age threshold was used to split the data into two liability classes for genetic analyses.
(PDF)

S4 Fig Gender differences in the music-related creative activities for three age groups. The frequencies within each age group are shown here for arranging, composing, NCNA and other creativity separately for males and females. The music-related creative activities are more common among music-experienced males than females in all three age groups (<20 years old, 20–55 years old and >55 years old).
(PDF)

S5 Fig Joint linkage and LD. The chromosomes are shown on the X-axis and results on the Y-axis. The results are given here as–log(p-value); the larger values denote stronger association. The best associations remain suggestive; significant results would rise above 8.
(PNG)

S6 Fig Regional association plot for arranging for chromosome 2. The joint linkage and LD p-values are given on the Y-axis as negative logarithms (Observed). The linkage disequilibrium between the best associated SNP, rs6531144, and the other SNPs are colour-coded according to their pairwise R-squared values (see the figure legend). The light blue curve shows the background recombination rate (HapMap). Genes in the region are shown at the bottom. From them, FAM49A with unknown function is on the same LD block as the associated SNPs.
(PDF)

S7 Fig Regional association plot for NCNA at chromosome 6. The joint linkage and LD p-values are given on the Y-axis as negative logarithms (Observed). The linkage disequilibrium between the best associated SNP, rs6531144, and the other SNPs are colour-coded according to their pairwise R-squared values (see the figure legend). The light blue curve shows the background recombination rate (HapMap). Genes in the region are shown at the bottom. From them, MAP3K7 is nearest of the associated SNPs.
(PDF)

S8 Fig Regional association plot for other creativity at chromosome 10q22.1. The joint linkage and LD p-values are given on the Y-axis as negative logarithms (Observed). The linkage disequilibrium between the best associated SNP, rs1417940, and the other SNPs are colour-coded according to their pairwise R-squared values (see the figure legend). The light blue curve shows the background recombination rate (HapMap). Genes in the region are shown at the bottom.
(PDF)

S9 Fig Regional association plot for other creativity at chromosome 2q31.1. The joint linkage and LD p-values are given on the Y-axis as negative logarithms (Observed). The linkage disequilibrium between the best associated SNP, rs4972527, and the other SNPs are colour-coded according to their pairwise R-squared values (see the figure legend). The light blue curve shows the background recombination rate (HapMap). Genes in the region are shown at the bottom.
(PDF)

S1 Table Questionnaire considering creativity. Here are the translations of the original Finnish questions (available from the authors) considering creativity. The questionnaire was available either through the Internet or on paper.
(PDF)

S2 Table Overrepresented functions and diseases in the gene sets suggestively associated in joint linkage and LD analyses. The best functions are listed here with related genes that were associated in joint LD and linkage analyses (Genes and # genes). Cancer, especially solid tumour, related functions were overrepresented with all phenotypes, but this is highly likely to be due to cancer being overrepresented in expression databases and thus it was not considered as a truly associated function.
(PDF)
