# Supplementary figures and images for: Creative Activities in Music – A Genome-Wide Linkage Analysis
Source: PLoS One. 2016 Feb 24;11(2):e0148679. doi: 10.1371/journal.pone.0148679 (PMC4766096; doi:10.1371/journal.pone.0148679)

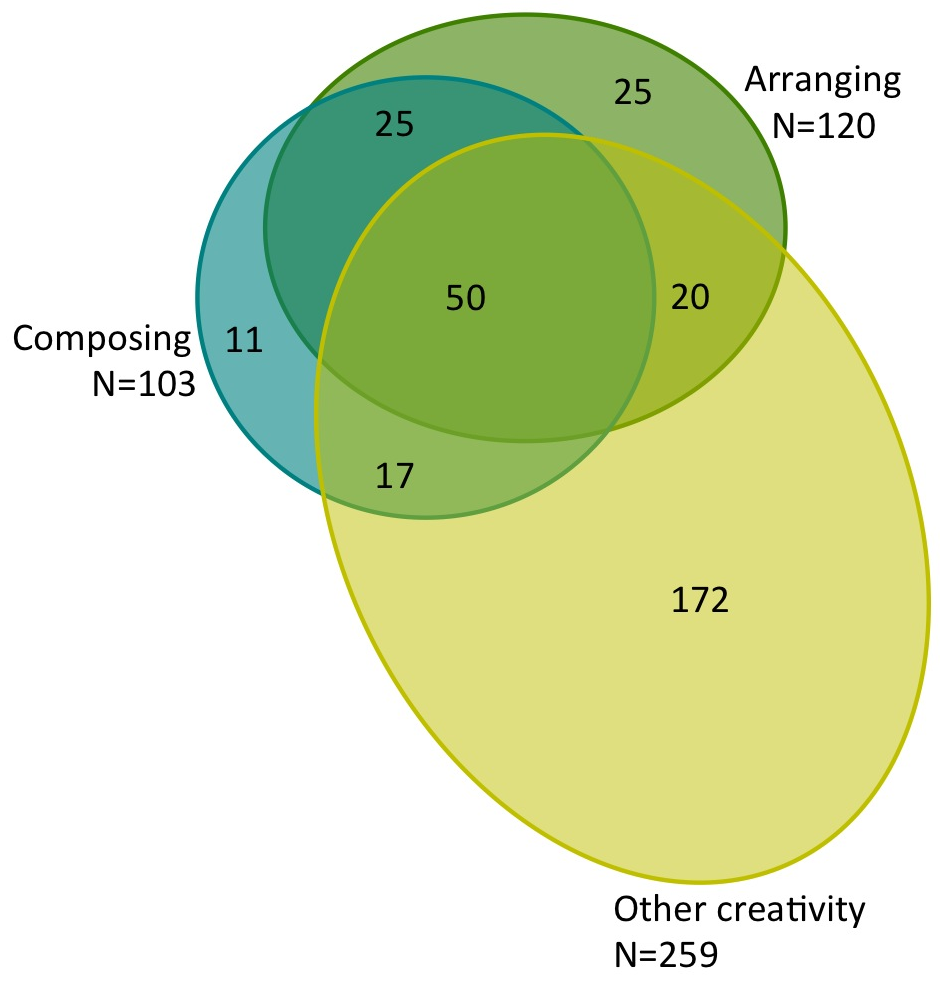

Supplement: S1 Fig — The number of the “yes” answers for composing, arranging and other creativity are shown here with overlap between the answers. Over half of the “yes” answers between arranging and composing overlapped. Overall, 320 individuals arranged, composed or considered themselves to be creative. (PNG) [file pone.0148679.s001.png]

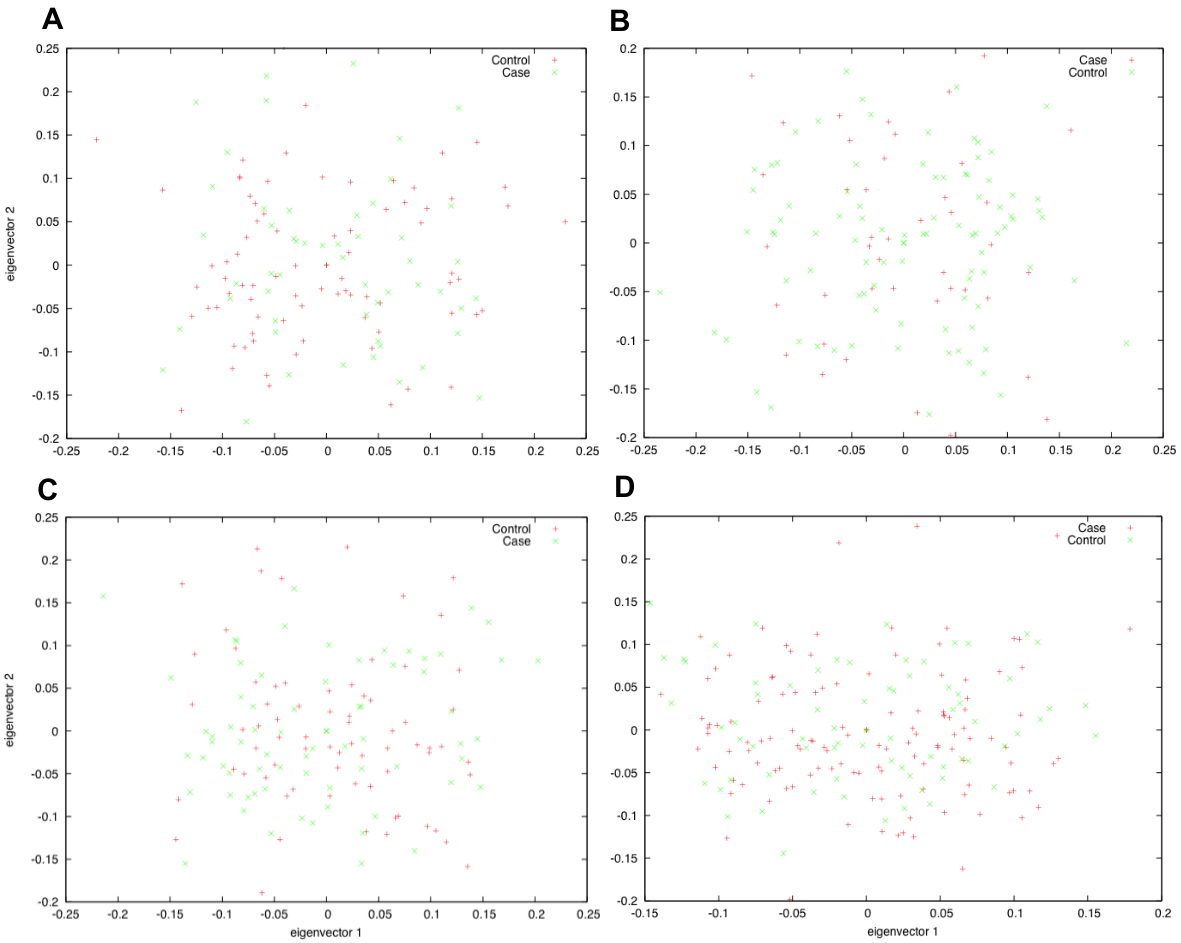

Supplement: S2 Fig — The population stratification was studied with principal component analysis using EIGENSOFT 6.0.1. Only unrelated individuals (founders or other unrelated individuals from each family) were used. Plots from the top two principal components are here shown for arranging (A), composing (B), NCNA (C) and other creativity (D). (PNG) [file pone.0148679.s002.png]

**A**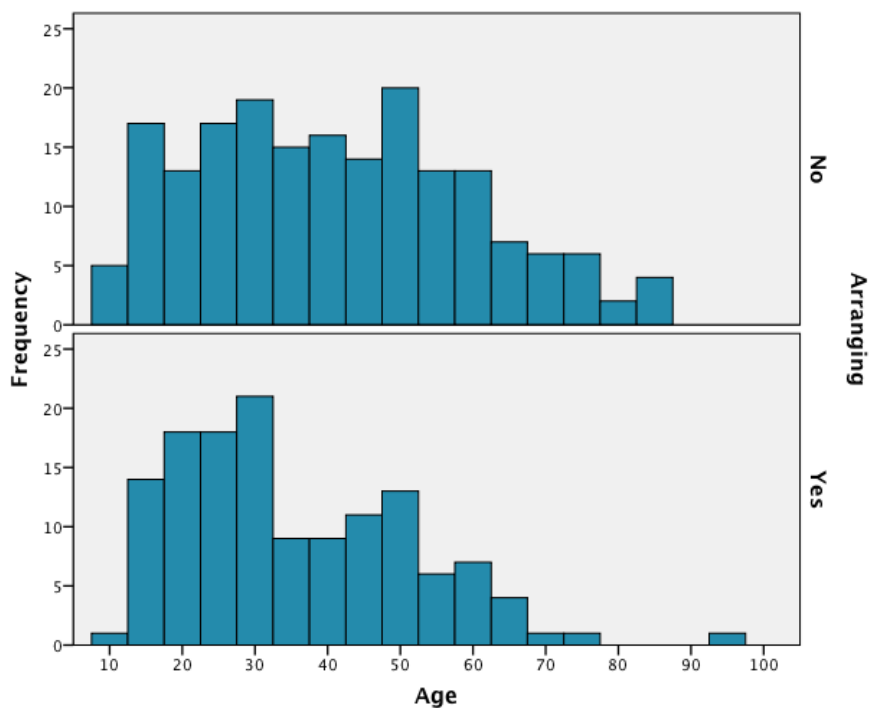**B**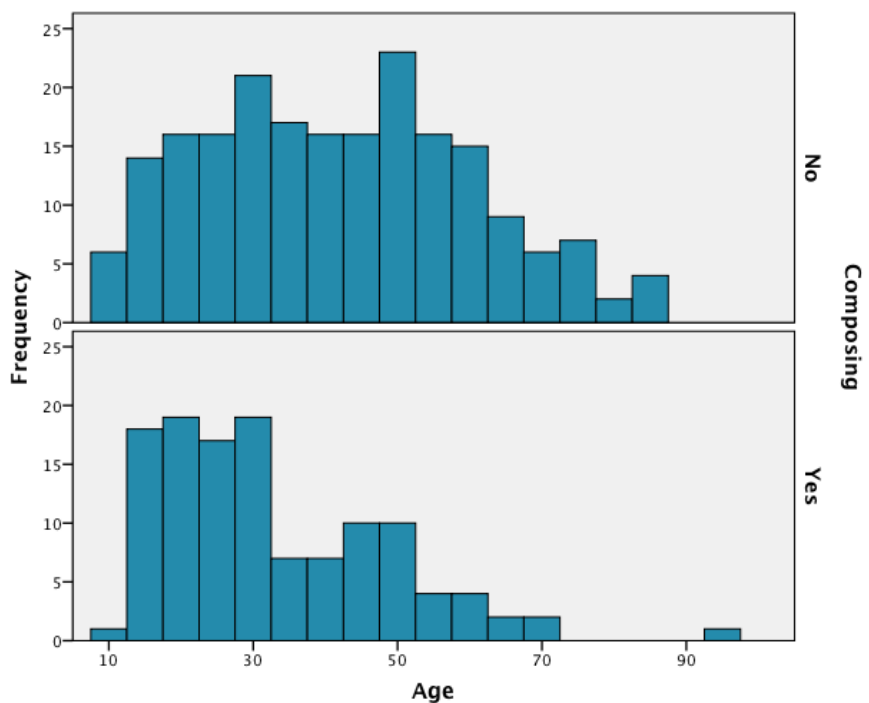

Supplement: S3 Fig — Histograms of age among active and passive subjects in arranging (A) and composing (B). Frequency of the individuals who compose or arrange drops after the age of 32. This age threshold was used to split the data into two liability classes for genetic analyses. (PDF) [file pone.0148679.s003.pdf]

Females

Males

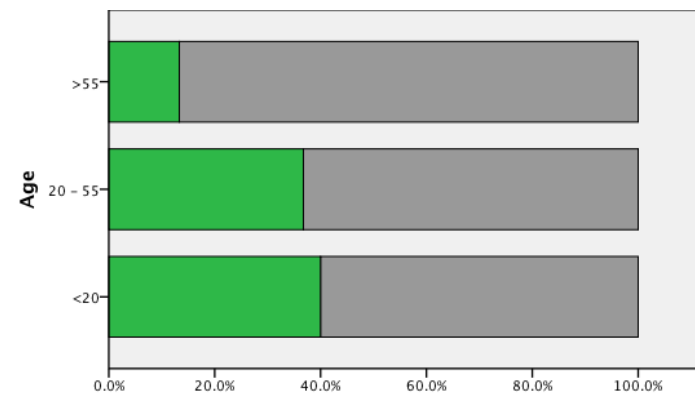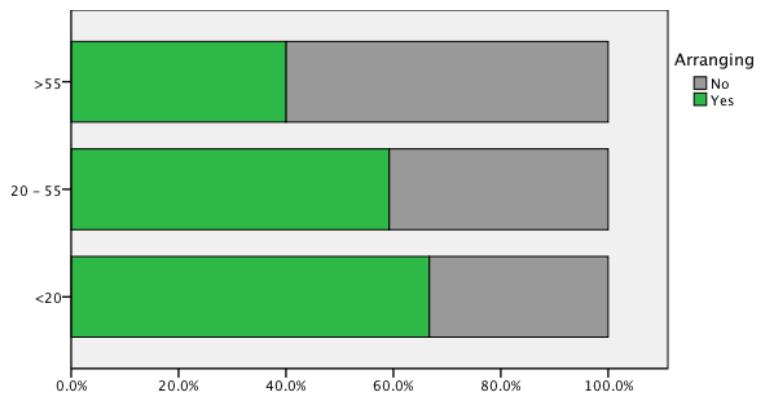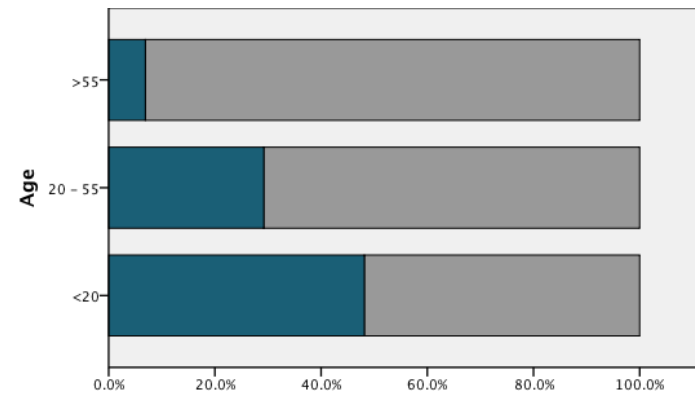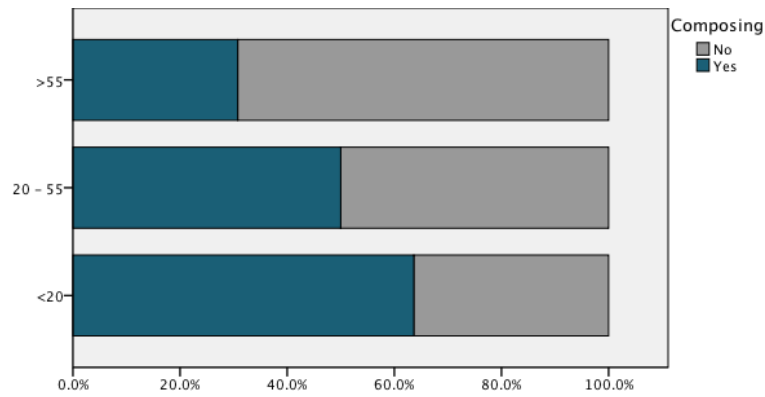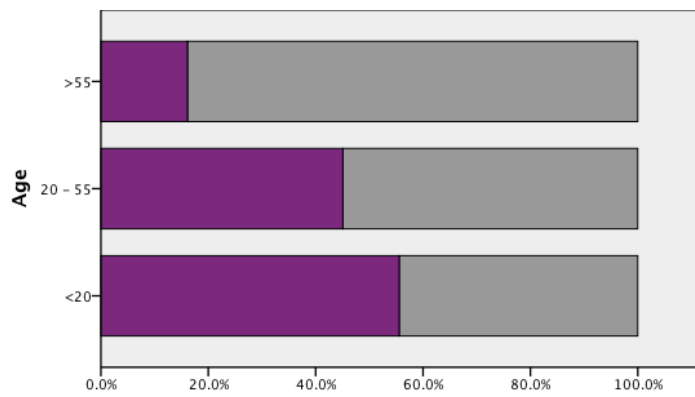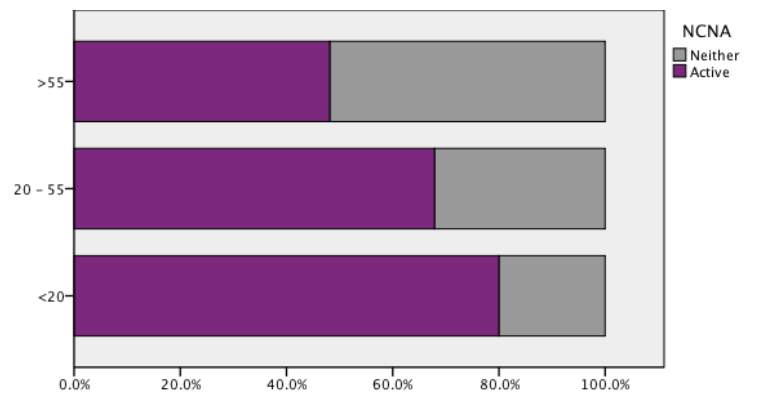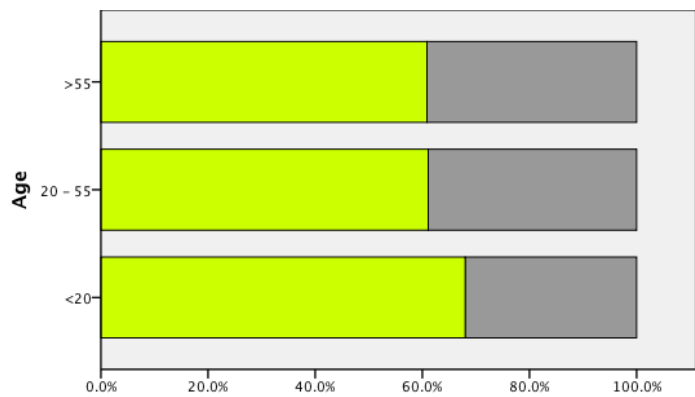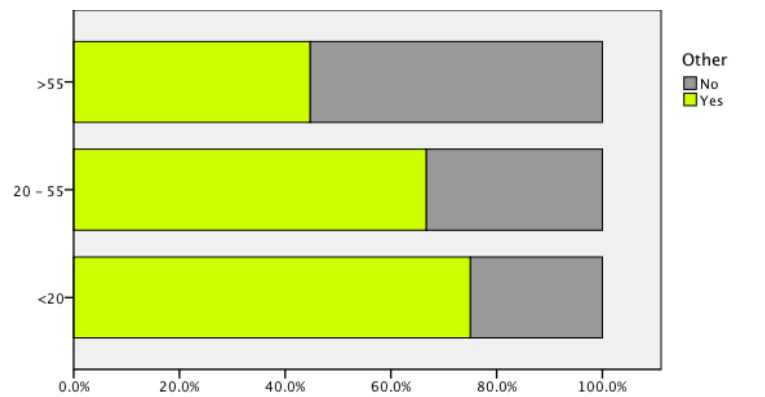

Supplement: S4 Fig — The frequencies within each age group are shown here for arranging, composing, NCNA and other creativity separately for males and females. The music-related creative activities are more common among music-experienced males than females in all three age groups (<20 years old, 20–55 years old and >55 years old). (PDF) [file pone.0148679.s004.pdf]

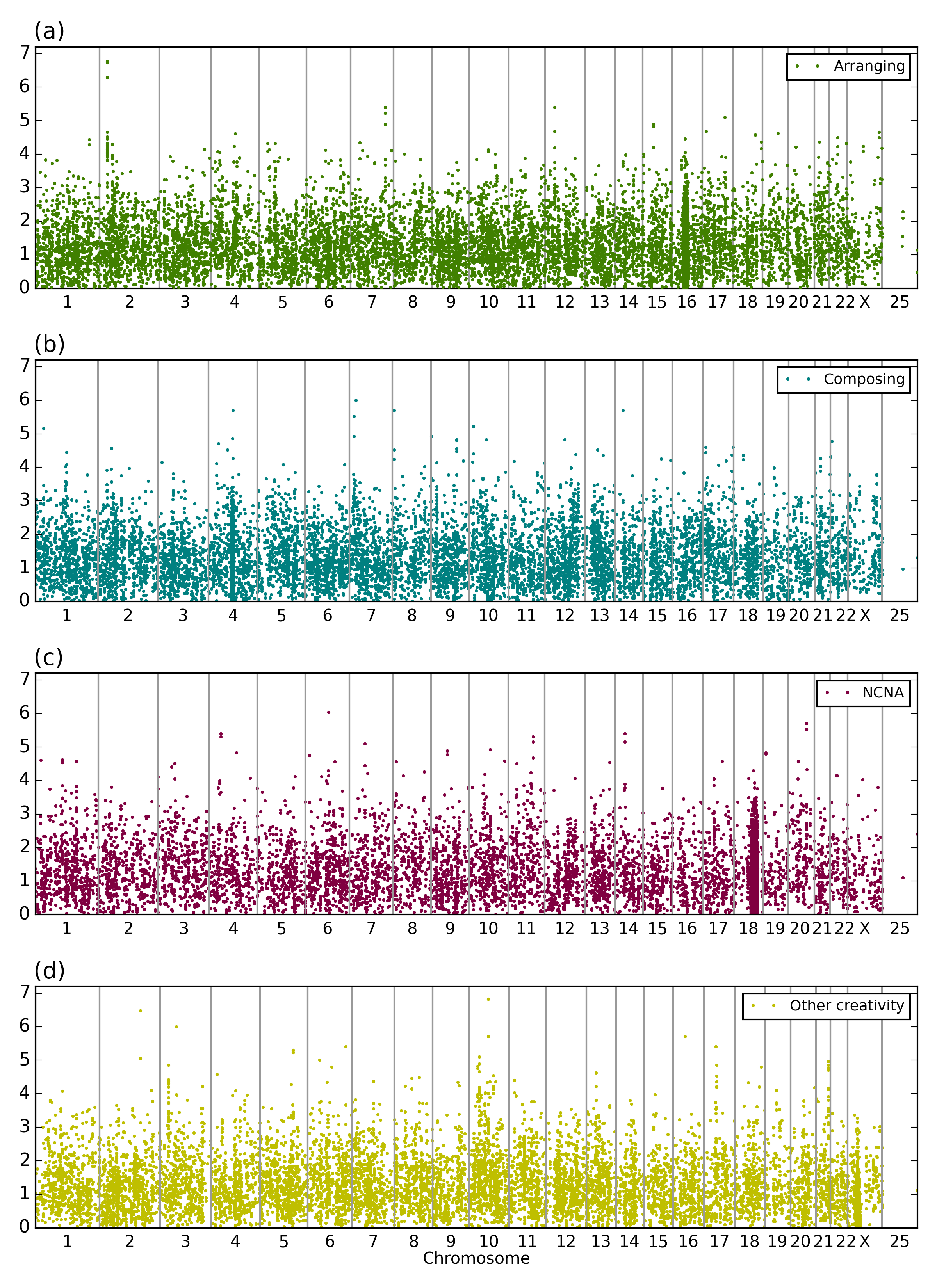

Supplement: S5 Fig — The chromosomes are shown on the X-axis and results on the Y-axis. The results are given here as–log(p-value); the larger values denote stronger association. The best associations remain suggestive; significant results would rise above 8. (PNG) [file pone.0148679.s005.png]

# Arranging

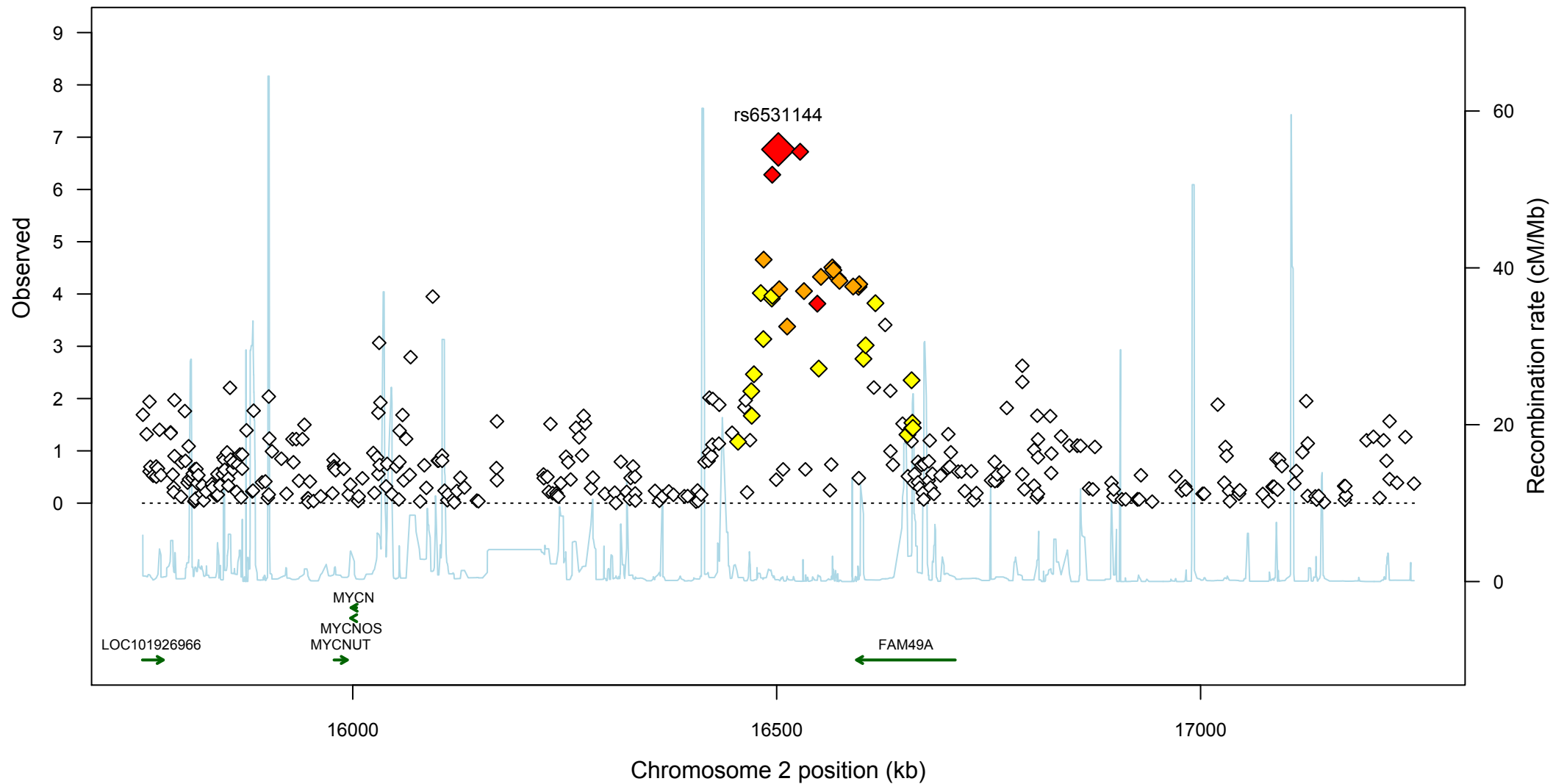

Supplement: S6 Fig — The joint linkage and LD p-values are given on the Y-axis as negative logarithms (Observed). The linkage disequilibrium between the best associated SNP, rs6531144, and the other SNPs are colour-coded according to their pairwise R-squared values (see the figure legend). The light blue curve shows the background recombination rate (HapMap). Genes in the region are shown at the bottom. From them, FAM49A with unknown function is on the same LD block as the associated SNPs. (PDF) [file pone.0148679.s006.pdf]

# NCNA

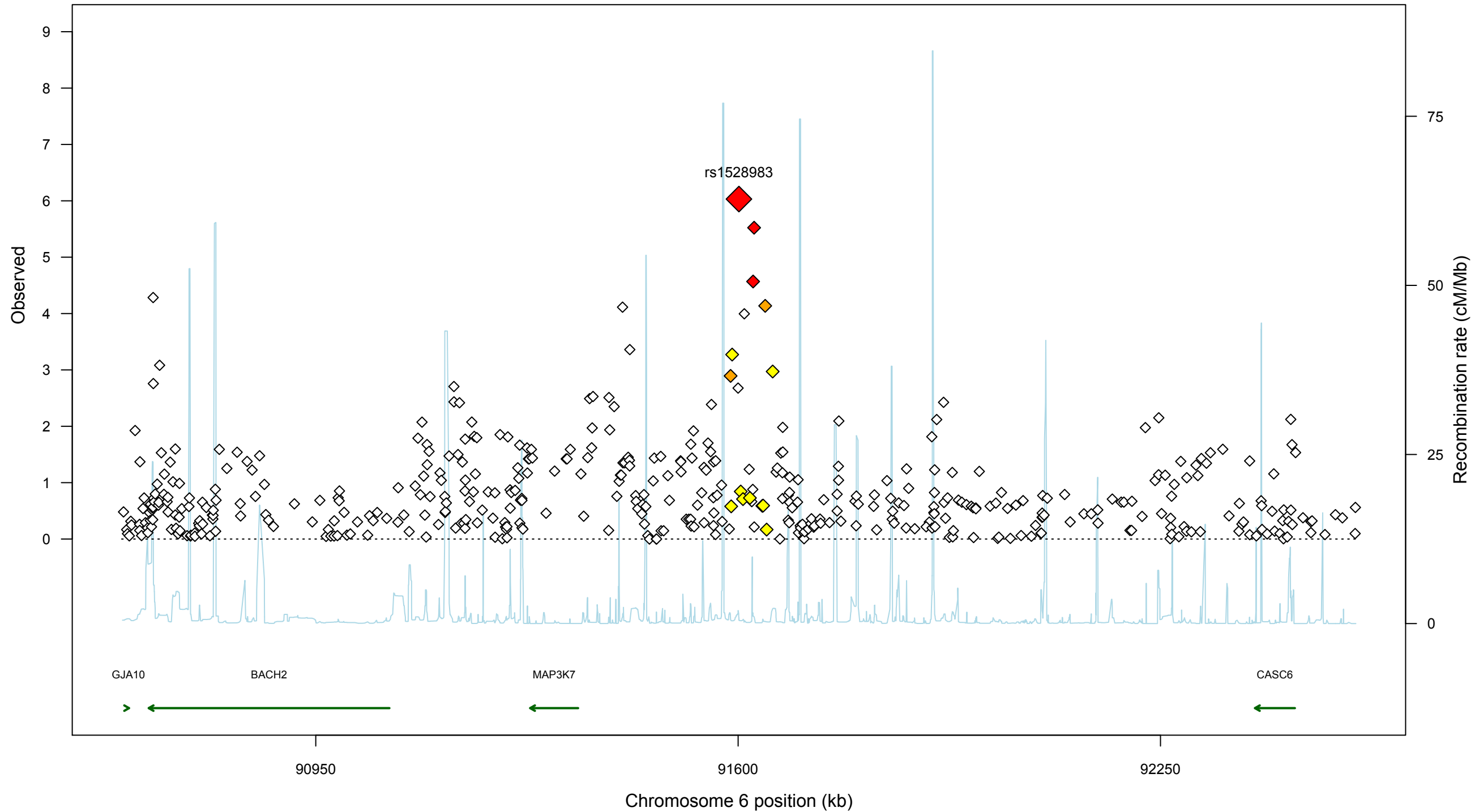

Supplement: S7 Fig — The joint linkage and LD p-values are given on the Y-axis as negative logarithms (Observed). The linkage disequilibrium between the best associated SNP, rs6531144, and the other SNPs are colour-coded according to their pairwise R-squared values (see the figure legend). The light blue curve shows the background recombination rate (HapMap). Genes in the region are shown at the bottom. From them, MAP3K7 is nearest of the associated SNPs. (PDF) [file pone.0148679.s007.pdf]

## Other creativity

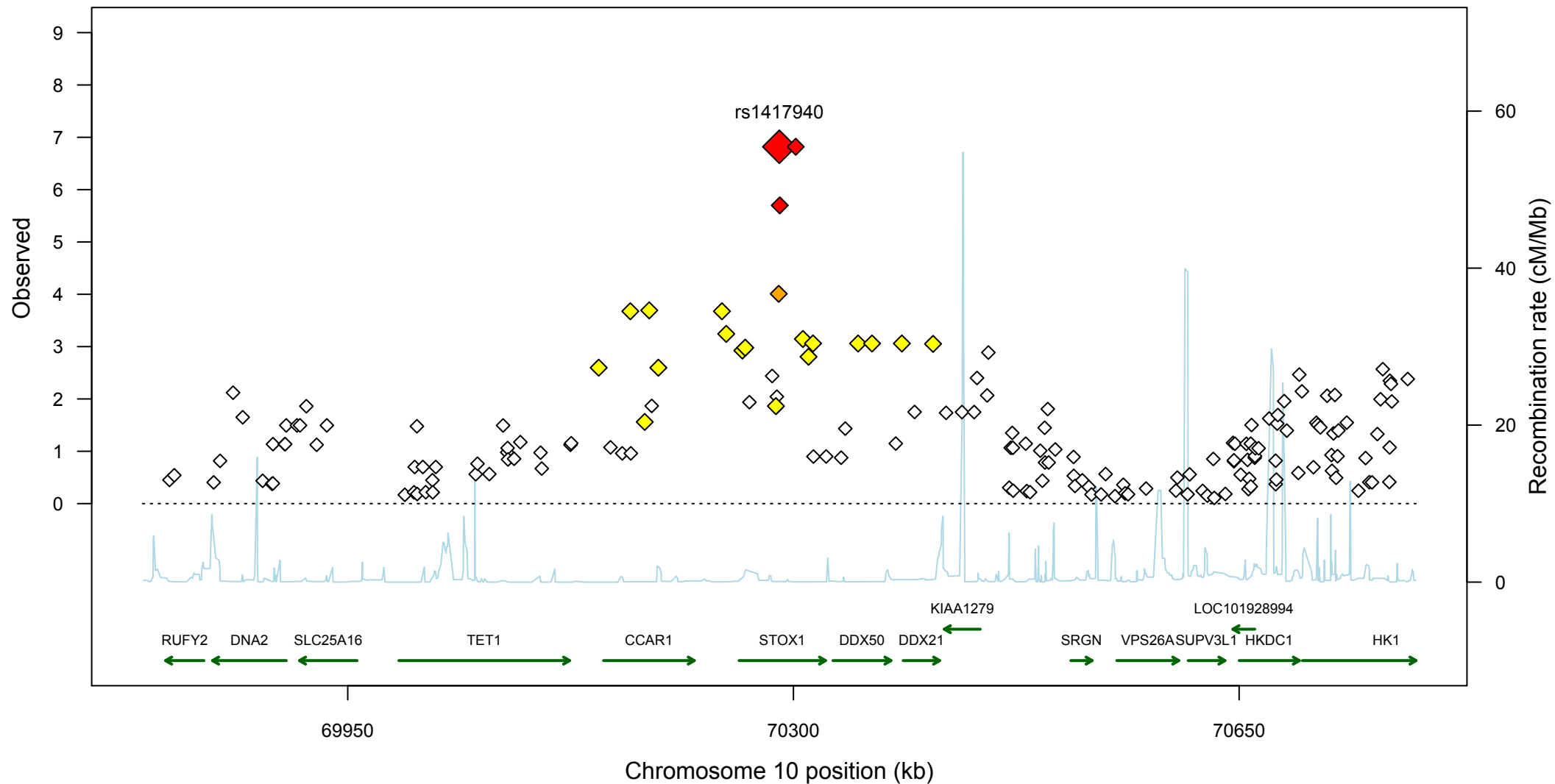

Supplement: S8 Fig — The joint linkage and LD p-values are given on the Y-axis as negative logarithms (Observed). The linkage disequilibrium between the best associated SNP, rs1417940, and the other SNPs are colour-coded according to their pairwise R-squared values (see the figure legend). The light blue curve shows the background recombination rate (HapMap). Genes in the region are shown at the bottom. (PDF) [file pone.0148679.s008.pdf]

## Other creativity

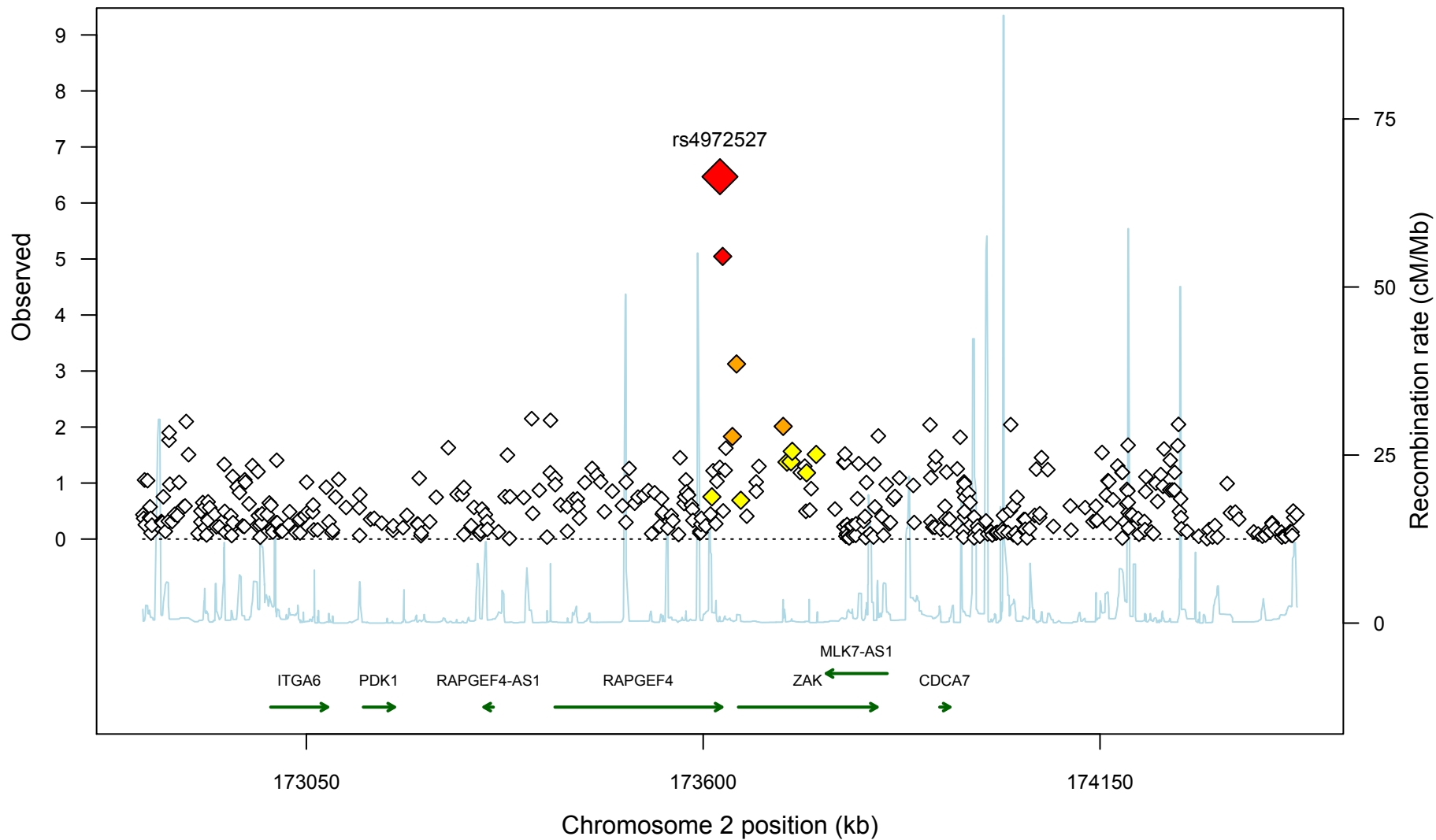

Supplement: S9 Fig — The joint linkage and LD p-values are given on the Y-axis as negative logarithms (Observed). The linkage disequilibrium between the best associated SNP, rs4972527, and the other SNPs are colour-coded according to their pairwise R-squared values (see the figure legend). The light blue curve shows the background recombination rate (HapMap). Genes in the region are shown at the bottom. (PDF) [file pone.0148679.s009.pdf]
