# Supplementary material for: Creative Activities in Music – A Genome-Wide Linkage Analysis
Source: PLoS One. 2016 Feb 24;11(2):e0148679. doi: 10.1371/journal.pone.0148679 (PMC4766096; doi:10.1371/journal.pone.0148679)
Supplement: S1 Table — Here are the translations of the original Finnish questions (available from the authors) considering creativity. The questionnaire was available either through the Internet or on paper. (PDF) [file pone.0148679.s010.pdf]

S1 Table. Questionnaire considering creativity.

Explanation	Question	Options
Composing refers here to creating new piece of music. It can be in any form: score, lead sheet, recording or some other form that can be shared with other people. It can also be of any length.	Do you compose?	[ ] Yes<br>[ ] No<br>[ ] Did before, but not anymore<br>[ ] Don't know
Arranging refers here to making new presentable versions of compositions in such a way that the original composition can be identified.	Do you arrange?	[ ] Yes<br>[ ] No<br>[ ] Did before, but not anymore<br>[ ] Don't know
Non-musical creativity refers here to visual, scientific, technical, physical and/or verbal creativity. For example drawing, writing and acting are usually creative. You can choose "very creative" even though you are very creative only in one field.	How creative do you consider yourself in non-musical creativity?	[ ] Very creative<br>[ ] Quite creative<br>[ ] Slightly creative<br>[ ] Not at all creative<br>[ ] Don't know
