# Supplementary material for: Creative Activities in Music – A Genome-Wide Linkage Analysis
Source: PLoS One. 2016 Feb 24;11(2):e0148679. doi: 10.1371/journal.pone.0148679 (PMC4766096; doi:10.1371/journal.pone.0148679)
Supplement: S2 Table — The best functions are listed here with related genes that were associated in joint LD and linkage analyses (Genes and # genes). Cancer, especially solid tumour, related functions were overrepresented with all phenotypes, but this is highly likely to be due to cancer being overrepresented in expression databases and thus it was not considered as a truly associated function. (PDF) [file pone.0148679.s011.pdf]

S2 Table. Overrepresented functions and diseases in the gene sets suggestively associated in joint linkage and LD analyses.

Diseases or Functions	p-value	Genes	# genes	Phenotype
Insulin-dependent diabetes mellitus	4.33E-24	ABCF1, ABCG1, ABHD16A, ACTA2, AIF1, APOM, ATAT1, BAG6, C6orf15, C6orf47, C6orf48, CBS/ LOC102724560, CDSN, CFB, COL27A1, DDR1, DDX39B, DHX16, DPCR1, EHMT2, FLOT1, GPANK1, GTF2H4, HCG22, HCP5, HLA-B,HLA-C, HSPA1A/ HSPA1B, HSPA1L, IFIT1B, IFIT2, IFIT3, LSM2, LST1, LTA, LY6G5B, LY6G5C, LY6G6C, LY6G6D, MCCD1, MDC1, MICA, MICB, MSH5, MUC21, MZB1,NEBL, NELFE, PCDH15, POU5F1, PRRC2A, PSORS1C1, SKIV2L, SLC44A4, STK19,TCF19,TNF, TNFSF8, TUBB, UBASH3A, VARS2,VWA7, ZBTB12	63	Other creat.
Systemic autoimmune syndrome	1,01E-16	ABCF1,ABCG1, ABHD16A, ACTA2, AIF1, APOM, ATAT1, BAG6, BLNK, C2, C4A/C4B, C6orf15, C6orf47, C6orf48, C9orf152, CBS/ LOC102724560, CCHCR1, CDSN, CFB, CLU, COL27A1, DDR1, DDX39B, DHX16,DPCR1,EGR2,EHMT2,EPHX2, FAS, FLOT1, GNL1, GPANK1, GTF2H4, HCG22, HCG27, HCP5, HLA-B, HLA-C,HSPA1A/ HSPA1B, HSPA1L,IFIT1,IFIT1B, IFIT2, IFIT3, LSM2, LST1, LTA, LTB, LY6G5B, LY6G5C,LY6G6C,LY6G6D, MBL2, MCCD1, MDC1, MICA, MICB, MSH5, MUC21,MX1,MZB1,NCR3, NEBL, NELFE, NFKBIL1, NRM, PCDH15,PLAC4, POU5F1, PRRC2A, PSORS1C1, PTK2B, SIL1, SKIV2L, SLC44A4, STK19, TCF19, TMEM173, TNF, TNFSF11, TNFSF15, TNFSF8 ,TUBB, TXN, UBASH3A, VARS, VARS2, VIM, VWA7, ZBTB12	90	Other creat.
Rheumatoid arthritis	8,08E-12	AIF1, APOM, ATAT1, BAG6, BLNK, C2, C4A/C4B, C6orf15, C6orf47, C9orf152, CCHCR1, CFB, CLU, DDR1, DDX39B, DHX16, DPCR1, EHMT2, EPHX2, GNL1, GPANK1, GTF2H4, HCG22, HCG27, HLA-C, HSPA1A/ HSPA1B, HSPA1L, LST1, LTA,LTB, LY6G5C, LY6G6C, LY6G6D, MBL2, MCCD1, MDC1, MICA, MSH5, NELFE, NFKBIL1, NRM, PCDH15, PLAC4,PRRC2A,PSORS1C1,PTK2B, SIL1, SKIV2L, SLC44A4, STK19,TMEM173,TNF, TNFSF11, TNFSF15, TXN ,VARS, VARS2,VIM, ZBTB12	59	Other creat.
Chronic inflammatory disease	9,56E-09	ACTA2, ADRA1A, AIF1, APOM, ATAT1, BAG6, BLNK, BMI1, C2, C4A/ C4B, C6orf15, C6orf47, C9orf152, CCHCR1, CFB, CLU, DDR1, DDX39B, DHX16, DPCR1, DSC1, EHMT2, EPHX2, FAS, GNL1, GPANK1, GTF2H4, HCG22, HCG27, HGF, HLA-C, HSPA1A/ HSPA1B, HSPA1L, LST1,LTA, LTB,LY6G5C, LY6G6C, LY6G6D, MBL2, MCCD1, MDC1, MICA, MSH5,NELFE, NFKBIL1, NRM, PBLD, PCDH15,PLAC4, PRRC2A, PSORS1C1, PTK2B, SIL1, SKIV2L, SLC44A4, STK19, TMEM173,TNF, TNFSF11, TNFSF15, TNFSF8, TXN, VARS, VARS2, VIM, ZBTB12	67	Other creat.
Mulitple sclerosis	1,65E-08	ADRA1A, BAG6, C4A/C4B, C6orf15, CDSN, DHX16,EGR1, EHMT2, EPSTI1,FAS,HCG22, HLA-E, IFIT1,MCCD1, MX1, NELFE ,POLE3, POU5F1, PRRC2A, PSORS1C1, RRM2B, TCF19, TNF	23	Other creat.
Acquired immunodeficiency syndrome	7,05E-08	C4A/C4B, C6orf48, CLU, FAS, HCP5, HLA-C, MICB, PSORS1C1, TNF	9	Other creat.
Malignant solid tumor	1.31E-07	*	372	NCNA
	1.85E-06		423	Composing
	1.94E-05		432	Arranging
	1.54E-05		427	Other creat.
Cleavage of collagen fiber	1.02E-06	MMP1, MMP13, MMP3, MMP8	4	NCNA
Cancer	1.52E-06	*	436	Composing
	2.77E-06		378	NCNA
	4.81E-06		448	Arranging
	9,30E-05			Other creat.
X linked autism	2.09E-06	MECP2, NLGN3, RPL10, TMLHE	4	Arranging
Abdominal adenocarcinoma	3.55E-06	*	228	NCNA
X-linked hereditary disease	4.01E-06	ABCD1, ADRA1A, AVPR2, BNIP3L, CLIC2, DKC1, EMD, F8, FLNA, G6PD, GDI1, GJB1, HCFC1, HDAC8, IKBKG, IL2RG, L1CAM, MECP2, MED12, NAA10, OPN1LW, OPN1MW, RAB39B, TAZ, TGFB1	25	Arranging

Development of multicellular organism	4.19E-06	*DRG1, EN2, HOXA1, HOXA10, HOXA11, HOXA4, HOXA9, LIF, MYCNOS, NEUROG3, OSM, PLXNA3, RPS4X, TP53, ZMYM3*	15	Arranging
Cleavage of glycoprotein	4.91E-06	*MMP1, MMP13, MMP3, MMP8, PCSK5*	5	NCNA
Cell death	8.83E-06<br>2.39E-05	*	157<br>159	Other creat.<br>Arranging
	6.29E-05		134	NCNA
Apoptosis	8.90E-06<br>9.73E-05	*	129<br>127	Other creat.<br>Arranging
Morphogenesis of embryonic skeleton	9.95E-06	*HOXA2, HOXA3, HOXA4, HOXA5, HOXA6, HOXA7, MEGF8, MYCN, WNT9A*	9	Arranging
Cell death of male germ cells	1.40E-05	*BCL6, DMRTC2, ETV5, HORMAD2, HOXA11, LIMK2, MOV10L1, OSBP2, PAFAH1B3, SHBG, TASP1, TP53, YBX2*	13	Arranging
Compartmentalization of B lymphocytes	1.48E-05	*LTA, LTB, TNF*	3	Other creat.
Proteolysis of Gelatin	1.49E-05	*MMP1, MMP10, MMP12, MMP13, MMP3, MMP7*	6	NCNA
Cell death of germ cells	1.53E-05	*AXL, BCL6, DMRTC2, ETV5, HORMAD2, HOXA11, LIF, LIMK2, MOV10L1, OSBP2, PAFAH1B3, SHBG, TASP1, TP53, YBX2*	15	Arranging
Quantity of rib	2.07E-05	*BMPER, HOXA10, HOXA11, HOXA4, HOXA5, HOXA6, HOXA9, SHH, TASP1*	9	Arranging
Unwinding of RNA	2.76E-05	*DDX21, DDX50, EIF4A1, EIF4A2*	4	Arranging
Patterning of rostrocaudal axis	2.81E-05	*HOXA1, HOXA10, HOXA11, HOXA2, HOXA3, HOXA4, HOXA5, HOXA6, HOXA7, HOXA9, SHH, WNT3A*	12	Arranging
Morphology of vertebrae	2.84E-05	*BMPER, DVL2, HOXA10, HOXA11, HOXA3, HOXA4, HOXA5, HOXA6, HOXA9, SHH, TASP1, TGFB1, WNT3A, WNT9A*	14	Arranging
Morphology of rhombencephalon	3.15E-05	*AXL,CLN3, EBF2, EN2, GABRB3, HOXA1, HOXA2, KHDRBS2, L1CAM, LIF, PATZ1, PLXNB2, TP53, UBE3A, WNT3A*	15	Arranging
Proliferation of B lymphocytes	3.65E-05	*ARHGEF1, AXL, BANK1,BCL6, BLNK, CD19, CD79A, GAPT, IL21R, IL27, IL2RG, IL4R, MYCN, PATZ1, PIK3AP1, PIM3, POU2F2, SHH, ST6GAL1, TGFB1, TNFSF13, TP53, WNT3A*	23	Arranging
Apoptosis of male germ cells	3.67E-05	*BCL6, DMRTC2, ETV5, HORMAD2, HOXA11, LIMK2, OSBP2, PAFAH1B3, SHBG, TASP1, TP53, YBX2*	12	Arranging
Brachydactyly mental retardation syndrome	3.84E-05	*GPC1, GPR35, HDAC4*	3	NCNA
Hypertrophy of connective tissue	5.11E-05	*HDAC4, MC4R, MMP3, NOG, STC1, WNT5A, XBP1*	7	NCNA
Killing of E. coli	5.22E-05	*DEFA1, DEFB1, DEFB103A/DEFB103B, TICAM1, WNT5A*	5	NCNA
Hallucinations	5.96E-05	*CCKAR, HTR6, NPAS3*	3	Composing
Transport of histamine	5.96E-05	*SLC22A1, SLC22A2, SLC22A3*	3	Composing
Demyelination of fiber tract	6.58E-05	*CXCR3, GAL3ST1, TGFB1*	3	Arranging
Cerebral malaria	6.77E-05	*C4A/C4B, CFB, LTA, TNF*	4	Other creat.
Cell death of gonadal cells	6.92E-05	*AXL, BCL6, DMRTC2, ETV5, HORMAD2, HOXA11, LIF, LIMK2, MOV10L1, OSBP2, PAFAH1B3, SHBG, TASP1, TGFB1, TP53, YBX2*	16	Arranging
Morphology of cells	7.02E-05	*	100	Arranging
Abnormal morphology of glossopharyngeal nerve	7.93E-05	*HOXA1, HOXA2, HOXA3, MEGF8*	4	Arranging
Dysmyelination	8.21E-05	*ABCD1, ADIPOQ, CD19, CTC1, CXCR3, EBF2, GAL3ST1, GJB1, GJC2, IKBKG, LIF, MLC1, TGFB1*	13	Arranging
Adenocarcinoma	8.46E-05	*	239	NCNA
Abnormal morphology of	8.76E-05	*AXL, CLN3, EBF2, EN2, GABRB3, HOXA1, HOXA2, KHDRBS2, L1CAM, LIF, PATZ1, TP53, UBE3A, WNT3A*	14	Arranging

rhombencephalon				
Necrosis	9.27E-05	*	121	Other creat.
Neutralization of Influenza A virus	9.44E-05	*DEFA1, DEFA5, DEFA6*	3	NCNA
Morphology of gonadal cells	9.54E-05	*AXL, CTDNEP1, DMRTC2, ETV5, FETUB, HORMAD2, HOXA10, LIMK2,LIPE, MNS1, OSBP2, PAFAH1B3, PLA2G3, SH2B1, SPEM1, TASP1,TP53,YBX2*	18	Arranging
Morphology of reproductive system	9.55E-05	*AXL, BMPR1B, BRAF, CITED1, CTDNEP1, DMRTC2, EBF2, ERF, ETV5, FETUB, G6PD, GAL3ST1, HORMAD2, HOXA10, HOXA11, IL2RG, LIF, LIMK2, LIPE, MNS1, MOV10L1, NUPR1, OSBP2, PAFAH1B3, PATZ1, PLA2G3, PLK2, SH2B1, SLC34A2, SLIT2, SPEM1, TASP1, TGFB1, TP53, YBX2*	35	Arranging

* List of associated genes is not shown for functions with 100 or more genes.
